# DAIRYdb: a manually curated reference database for improved taxonomy annotation of 16S rRNA gene sequences from dairy products

**DOI:** 10.1186/s12864-019-5914-8

**Published:** 2019-07-08

**Authors:** Marco Meola, Etienne Rifa, Noam Shani, Céline Delbès, Hélène Berthoud, Christophe Chassard

**Affiliations:** 1 Agroscope, Competence Division Methods Development and Analytics, Research Group Fermenting Organisms, Schwarzenburgstrasse 161, Bern, 3003 Switzerland; 2 Université Clermont Auvergne, INRA, VetAgro Sup, UMRF, 20 côte de Reyne, Aurillac, 15000 France

**Keywords:** Microbiome, Taxonomy annotation, OTU classification, 16S, Database, Accuracy, Dairy, Cheese, Milk, Whey, Teat, Starter

## Abstract

**Background:**

Reads assignment to taxonomic units is a key step in microbiome analysis pipelines. To date, accurate taxonomy annotation of 16S reads, particularly at species rank, is still challenging due to the short size of read sequences and differently curated classification databases. The close phylogenetic relationship between species encountered in dairy products, however, makes it crucial to annotate species accurately to achieve sufficient phylogenetic resolution for further downstream ecological studies or for food diagnostics. Curated databases dedicated to the environment of interest are expected to improve the accuracy and resolution of taxonomy annotation.

**Results:**

We provide a manually curated database composed of 10’290 full-length 16S rRNA gene sequences from prokaryotes tailored for dairy products analysis (https://github.com/marcomeola/DAIRYdb).

The performance of the DAIRYdb was compared with the universal databases Silva, LTP, RDP and Greengenes. The DAIRYdb significantly outperformed all other databases independently of the classification algorithm by enabling higher accurate taxonomy annotation down to the species rank. The DAIRYdb accurately annotates over 90% of the sequences of either single or paired hypervariable regions automatically.

The manually curated DAIRYdb strongly improves taxonomic annotation accuracy for microbiome studies in dairy environments. The DAIRYdb is a practical solution that enables automatization of this key step, thus facilitating the routine application of NGS microbiome analyses for microbial ecology studies and diagnostics in dairy products.

**Electronic supplementary material:**

The online version of this article (10.1186/s12864-019-5914-8) contains supplementary material, which is available to authorized users.

## Background

The exploration of microbial communities has experienced a boost during the last decade with the advent of next-generation sequencing (NGS) technologies [1]. Previously undetectable micro-organisms in soils [2], water [3, 4], airborne [5, 6], snow [7], ice [8], food [9], human gut [10–12] etc. could be unravelled at an unprecedented depth and resolution. Numerous studies have been published describing microbial community structures in various environments, often correlating their dynamic changes over time or space by means of the 16S rRNA gene (16S) [13–15].

First microbiome studies using the 16S were based on fingerprinting techniques, such as Denaturing Gradient Gel Electrophoresis (DGGE), Terminal Restriction Fragment Length Polymorphism (T-RFLP) or Length Heterogeneity Polymerase Chain Reaction (LH-PCR) sometimes in combination with Sanger sequencing of the 16S to identify populations of interest. While Sanger sequencing delivered almost the complete 16S at good quality, the throughput was low due to the high workload, preventing researchers to unravel the full array of microbial diversity within a sample [16].

NGS has increased the sequencing depth, uncovering also low abundant micro-organisms, thus overcoming the limitations of Sanger sequencing. The higher sequencing depth of NGS, however, was obtained at the expense of read length, therefore limiting the sequencing to only few hyper variable regions (HVR). The short size of the resulting reads strongly reduces their resolution, while increasing the risk of taxonomic miss-annotation or ambiguous taxonomic classification. The need for trustworthy classification of very short 16S sequences covering only one to three HVR remains a crucial step to obtain robust and accurate taxonomic classification in modern microbiology [17].

Microbiome studies in dairy products are particularly affected by limitation in taxonomic annotation. Dairy environments are highly selective and thus often characterized by only few abundant genera belonging to the lactic acid bacteria (LAB). Therefore, complete microbial biodiversity in dairy products is only visible at species or even strain level, which makes taxonomic annotation at species level crucial for any diagnostics or microbial ecology study. Moreover, short fragment strategies, on single HVRs or HVR pairs, often fail to reliably assign the correct taxonomy at the species level. It is therefore of paramount importance to select the right HVRs to maximize taxonomy resolution in a given environment, especially when differences are visible at species level only.

In recent years, numerous classification algorithms have been developed and optimized to accurately annotate operational taxonomic units (OTUs) or amplicon sequence variants (ASVs) from short reads. Those classification tools have been developed for 16S and other genes based on different mathematical models, such as e.g., k-mer, Bayesian, Hidden Markov-Monte-Carlo model (HMM) etc. The Basic Local Alignment Search Tool (Blast) has long been the gold standard for sequence comparison and annotation [18]. More 16S specific taxonomy predictors have been developed, including RDP Naive Bayesian Classifier (NBC) [19], a naive Bayesian Classifier based on k-mers, GAST [20], MEGAN [21], Metaxa2 [22], riboFrama [23], SPINGO [24], PROTAX [25], SINTAX [26], DynamiC [27], Humidor [28], MAPseq [29], microclass [30], q2-feature-classifier [31], IDTAXA [32] and other tools implemented in the most current 16S pipelines like mothur [33], Qiime v1 [34], Qiime v2 [35] and FROGS [36].

Although classification prediction algorithms have strongly improved, manually curated databases containing only authoritative full-length 16S sequences from type strains and cultivated reference strains can potentially compensate the limitations of short read sequences annotations by means of sophisticated algorithms. To date, three main independent universal repositories dedicated to universal 16S sequences from prokaryotes are widely used: Silva, The Ribosomal Database Project (RDP), and Greengenes (GG) [37].

Silva is the universal 16S repository with the highest number of sequences. The latest release of Silva SSU/LSU 132 (www.arb-silva.de) contained 6’073’181 16S sequences of at least 300 bp, with 2’090’668 good quality sequences with at least 900 bp length [38–40]. Taxonomic rank information of Silva and Living Tree Project (LTP) are based on the Bergey’s Taxonomic Outlines and the List of Prokaryotic Names with Standing Nomenclature (LPSN) [41]. Minimal training sets, such as the SSU Ref NR 99 or the LTP [42], offer a reduced number of sequences for faster classification but still covering the broadest currently known biodiversity.

The second biggest repository, the Ribosomal Database Project (RDP Release 11, Update 5; http://rdp.cme.msu.edu) [43], contained at the time of writing 3’356’809 16S sequences from the International Nucleotide Sequence Database Collaboration (INSDC) [44]. The nomenclature is based on the Bacterial Nomenclature Up-to-Date and the taxonomic rank information on the Bergey’s Manual.

Greengenes v13 _5 [45] contains 1’800’000 quality filtered 16S sequences. Classification nomenclature is based on automatic de novo tree construction and rank mapping with the NCBI Taxonomy database [46]. Although frequently used in community studies together with Qiime [34], the last update dates back to 2013 with no indication for an imminent update.

Taxonomic classification of the 16S is not trivial and requires both familiarity with prokaryotic phylogeny and often manual intervention due to poor annotation of the OTUs or by the available 16S databases [47]. Fast and accurate, thus automatized classification of the OTUs is not yet possible at the biologically most significant species rank due to the short sequence fragments and the absence of food-dedicated, thoroughly curated 16S databases. Manually curated databases are of paramount importance to improve reproducibility, speed during the bioinformatics process of microbiome studies, and communication between researchers [48]. Previous studies have highlighted the importance of high-quality data for improving the classification of the obtained OTUs [17, 49, 50]. Although universal 16S databases cover vast prokaryotic biodiversity, they often fail to guarantee accurate classification to the species rank for sequences obtained from a highly studied environment, such as dairy products. In fact, annotation accuracy at lower taxonomic ranks increases with a standard training set encompassing only full-length and good quality representative sequences innate to the investigated environment [17, 48, 50–52].

Here we present a comprehensive reference database, DAIRYdb (Database, Agroscope, Inra, Ribosomal, accuracY), for 16S OTUs classification of next generation sequencing (NGS) reads from dairy products. The main goal was to develop a dedicated database that allows researchers to accurately and automatically annotate short reads of 16S down to the species level. The performance of the DAIRYdb was compared with the universal databases Silva, RDP, Greengenes and LTP using three predictors based on different algorithms and programming languages [53], such as Blast+ [54], Metaxa2, [22, 55], and SINTAX [26]. Manual curation of the database and its restriction to the biodiversity expected in dairy products strongly improves accuracy and reproducibility of phylogenetic classification at all taxonomic ranks.

DAIRYdb is publicly available at https://github.com/marcomeola/DAIRYdb and can be integrated in any classification prediction tool that allows adaptation of customized databases, such as Blast+, Metaxa2, SINTAX, IDTAXA and FROGS.

## Results and discussion

### Construction

**Fig. 1:**
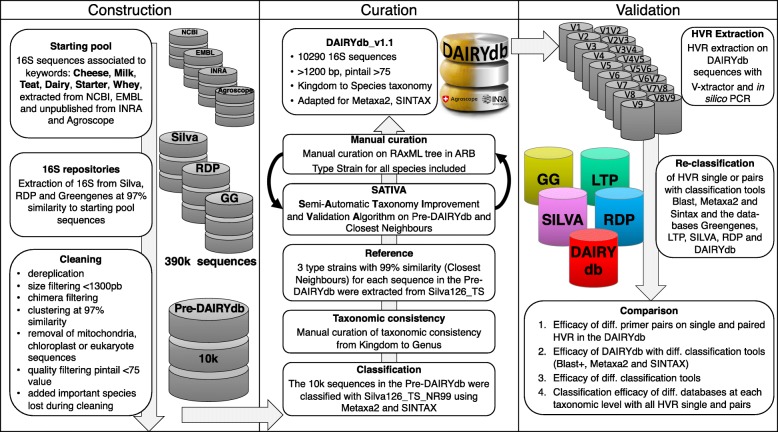
Development of the DAIRYdb consisted in three main steps: construction, curation and validation. For construction, dairy products specific 16S sequences were retrieved from Silva, RDP and Greengenes using Genbank NCBI, EMBL, Agroscope and INRA sequences. Curation was performed based on the cross-validation results from the leave-one-out test of SATIVA and highly iterated RAxML tree, followed by manual curation of taxonomic assignment and consistency throughout all taxonomic ranks, with a particular focus on singleton taxons with no reference sequence. Validation was performed comparing taxonomy annotation accuracy of single and HVR pairs by the five databases (Greengenes 13.8, LTP version, Silva 128 NR99, RDP version and DAIRYdb)

The 16S sequence database of dairy products DAIRYdb was constructed using a set of over 390’000 sequences associated to the selected keywords (cheese, milk, teat, dairy, starter, whey) deposited in NCBI GenBank and ENA/EMBL, as well as sequences with 97% average nucleotide identity (ANI) from Silva, RDP and Greengenes (Fig. 1). About 10’000 best quality reference sequences were retained after filtering based on sequence length (>1300 bp), quality (pintail >75) and potential chimeras. Finally, 16S sequences of important species from cheese and dairy environments [56, 57] lost during the clustering were added subsequently. The final number of 16S sequences consequently reached 10’290.

**Fig. 2:**
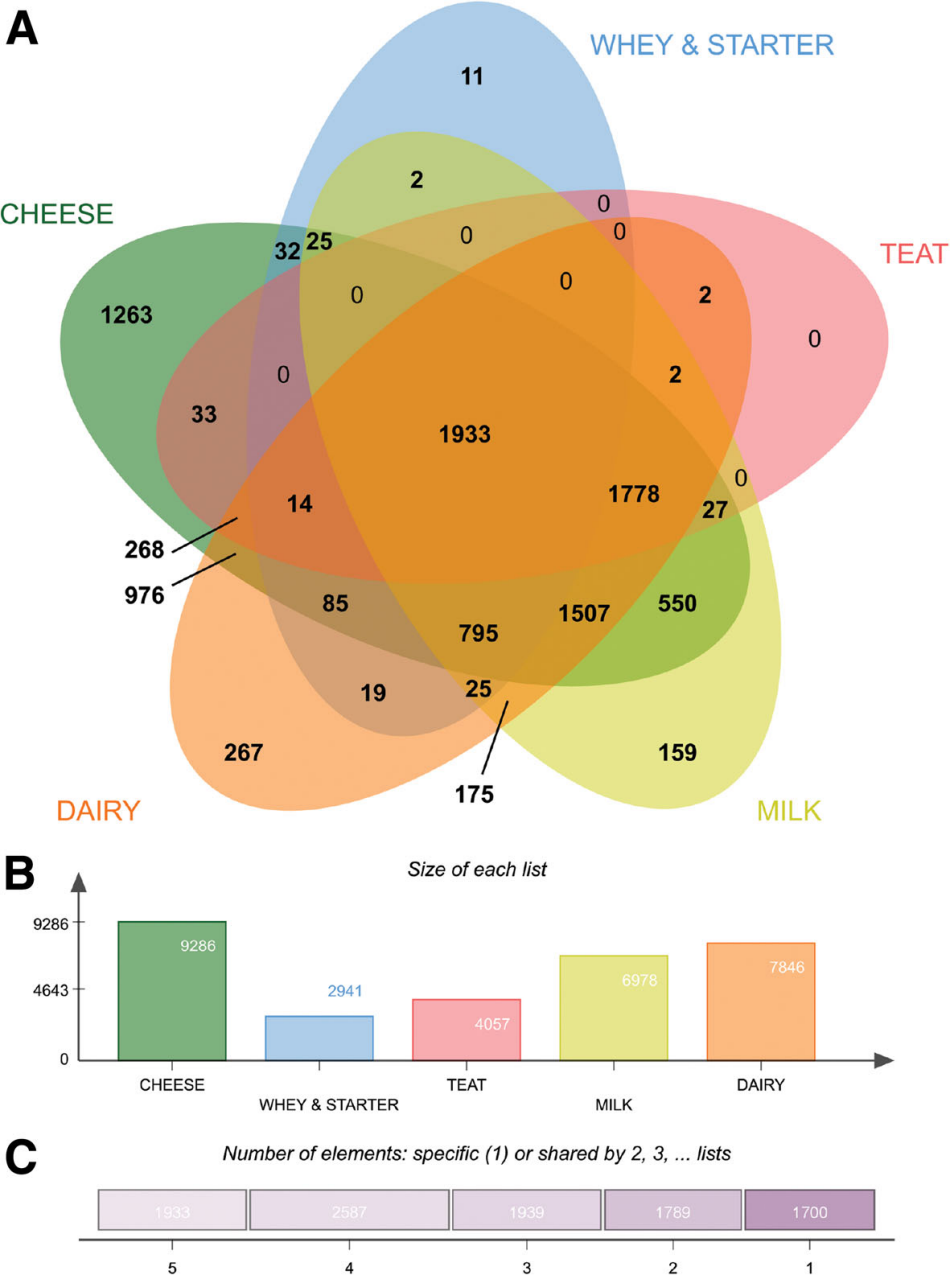
Origin of sequences in the DAIRYdb. **a** Five-factors Venn diagram comparing the origins of the sequences (9’948) retrieved from the public repositories Genbank NCBI and EMBL associated to the keywords “cheese”, “dairy”, “milk”, “teat” and “whey/starter”. About 12.7% (1’263) sequences were only detected in cheese and 15.1% (1’507) were detected in all three cheese, milk and dairy environments. **b** Total number of sequences associated to a particular keyword. **c** Number of sequences shared by 1 to 5 keywords. About 19.4% (1’933) sequences were detected in all 5 keywords, while 17.1% (1’700) sequences were unique to one keyword

The observed distribution among the different key words might reflect the unequal distribution of microbiome studies predominantly performed on cheese, dairy and milk samples, as compared to teats and whey. About 1933 sequences of the DAIRYdb were shared among all keywords (Fig. 2a) and 1778 were shared among the keywords dairy, cheese and milk. In fact, the majority of the sequences composing the DAIRYdb were linked to those three keywords (Fig. 2b). Altogether, 1’700 sequences were associated to just one keyword, with most of the sequences shared by four keywords (Fig. 2c).

### Curation

During the first step of data curation, the sequences were taxonomically annotated with Silva by means of SINA [58]. The resulted annotation at all taxonomic ranks underwent a first manual check and cleaning for taxonomic inconsistencies through cross-comparison with the other members of the same taxonomic rank in a phylogenetic tree. No taxonomic overlaps comparable to other databases are present in the DAIRYdb, where different species of the same genus fall under different taxonomic lineages [48]. A maximum of three closest neighbour type strains (CN) with authoritative taxonomy from Silva sharing 99% global sequence similarity to each sequence in the DAIRYdb were added to the 10’290 sequences in the DAIRYdb as reference during the curation process and removed at the end of the curation process. The maximal number of lowest common ancestors (LCA) with an authoritative taxonomy strongly improved the curation process with the Semi-Automatic Taxonomy Improvement and Validation Algorithm (SATIVA) increasing robustness of the proposed changes of miss-annotated environmental sequences within the DAIRYdb [59].

By using only near full-length and curated 16S from type strains as reference sequences, we were able to validate and correct the taxonomy annotation where necessary. The SATIVA results were inspected and taxonomy manually curated using a highly iterated phylogenetic tree. The approach used during the manual curation broadly follows the rationale described in detail in a recently published study [48]. Taxonomy annotations from authoritative type strain sequences were used as reference for the environmental sequences in the tree. For ranks at which no taxonomic annotation was possible with certainty due to the lack of authoritative type strains within the same clade (*i.e.*, commonly labelled “unknown”, “uncultured” etc. in universal databases), the *lowest common rank* (LCR) [52] was used down to the species rank with the addition of the unclassified rank. As an example, a sequence assigned to the LCR, the genus *Sporichthya*, was named at species rank *Sporichthya_Species*. This approach avoids the merging of abundance values from different unknown species to biologically uninformative groups, thus improving communication among scientists [60].

**Fig. 3:**
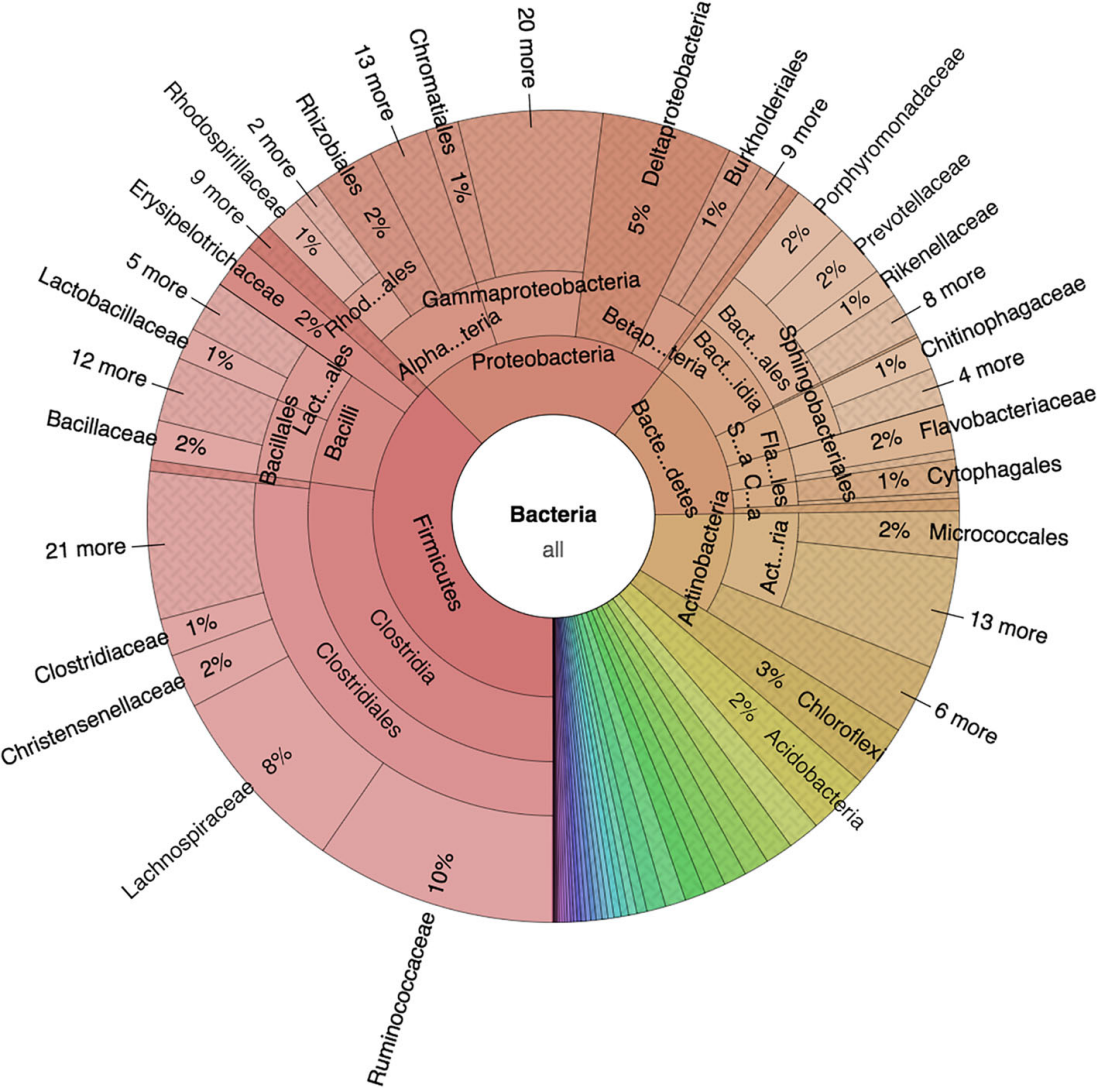
Complete microbial diversity present in the DAIRYdb. Prokaryotic biodiversity in the DAIRYdb is represented by 2 kingdoms, 47 phyla, 136 classes, 249 orders, 463 families, 1’757 genera and 4’030 unique species-like groups. The most represented phylum is Firmicutes (37% of all sequences), followed by the Proteobacteria (22%), Bacteroidetes (14%), Actinobacteria (9%), Chloroflexi (2%), Acidobacteria (2%), Archaea (1%) and 34 other minor phyla

DAIRYdb version 1.1 contains 2 kingdoms (Bacteria and Archaea), 47 phyla, 136 classes, 249 orders, 463 families, 1’757 genera and 4’030 unique species-like groups/species complexes (Fig. 3, Additional files 1 and 2). The *Firmicutes* is the predominant phylum with 37% of all sequences, followed by the *Proteobacteria* (22%), *Bacteroidetes* (14%), *Actinobacteria* (9%), *Chloroflexi* (2%), *Acidobacteria* (2%), Archaea (1%) and 34 other minor phyla. The 1% of Archaea is subdivided into *Euryarchaeota* (74%), *Crenarchaetoa* (13%), *Thaumarchaeota* (9%), *Woesearchaeota* (3%) and others (1%). Altogether, the DAIRYdb was able to capture the diversity of known taxa expected to occur in dairy products. Increasing number of whole genome sequences (WGS) will most likely lead to a replacement of incomplete 16S sequences in the DAIRYdb by full-length sequences that cover all HVRs.

The cheese microbiome is often dominated by few phylogenetically closely related species of LAB belonging to a few genera (*e.g.*, *Lactobacillus*, *Lactococcus*, *Leuconostoc* and *Streptococcus*) [9]. Therefore, special attention was put into the manual curation of the DAIRYdb sequences at species rank. Despite the genotypic and phenotypic characteristics of the most common LAB in cheese are extensively studied and described, several controversies regarding the nomenclature of some keystone species still remain unsolved, such as for the species *Lactococcus lactis* subsp. *lactis* and *Lactococcus lactis* subsp. *cremoris* [61]. It still is unresolved whether *Streptococcus thermophilus* is a species on its own or a subspecies of *Streptococcus salivarius* [62–64]. The DAIRYdb is composed of sequences retrieved from the Silva database along with their respective taxonomy, which was manually inspected for nomenclature hierarchy conflicts based on the phylogenetic position within the tree. However, some conflicting annotations of the same sequence were detected between the Silva taxonomy and the Bacterial Diversity Metadatabase, such as the species assignment of the type strain sequence Accession AB008205, which is labelled as *L. casei* in Silva and *L. paracasei* in Bacterial Diversity Metadatabase (BacDive) [65]. For the reference sequences of the most crucial species, bacterial names listed in the actual “List of prokaryotic names” according to BacDive were used. However, further disagreements between Silva and BacDive cannot be completely excluded. Moreover, some crucial genera in dairy products may undergo a radical genome-based relabelling in the future to create more homogeneous clusters [64].

Different approaches were applied on impure taxa, *i.e.* taxa that overlap in the tree despite being assigned to different nomenclature [48] in the universal databases. For instance, for the genera *Escherichia* and *Shigella*, Silva, LTP and RDP use the combined genus name *Escherichia–Shigella* but retain well-established species names, such as *Escherichia coli*. Differently, Greengenes leaves their sequences unclassified at ranks below the family *Enterobacteriaceae* [48]. The different taxonomic nomenclature references used by the three databases have an impact on revisions to resolve conflicts with sequence-based phylogenies and the labelling of new candidate groups identified in environmental sequences. However, discussion on the taxonomic inconsistencies and limitations of the universal databases (Silva, LTP, RDP and Greengenes), which the DAIRYdb was compared with, goes beyond the scope of this study and was extensively discussed elsewhere [48, 52].

The DAIRYdb will undergo regular updates in accordance to update on bacterial nomenclature [64], integrating the novelties or correcting the changes. Finally, the inclusion of full-length and high-quality 16S sequences from reference type strains leads to a more robust and confident taxonomic classification [49].

### Validation

At present, only short read sequences can be obtained from the most common amplicon NGS sequencers with at least 99% read quality and up to 600 bp in read length (Illumina MiSeq, Ion Torrent S5). Although long reads sequencing technology, such as PacBio and Oxford Nanopore, are steadily improving read quality, they are not yet routinely used for amplicon metabarcoding studies. Therefore, performance of the DAIRYdb was evaluated on short read sequences spanning over either a single HVR or HVR pairs.

**Fig. 4:**
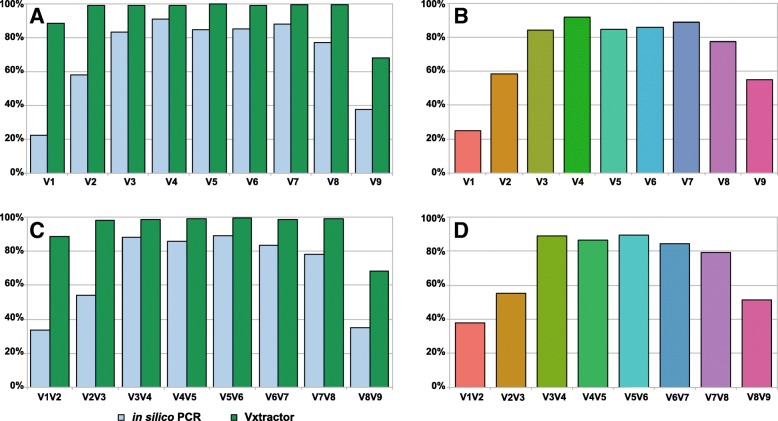
Presence and extraction efficiency of all HVR in the sequences of the DAIRYdb. Single HVR (**a**) and (**b**) and HVR pairs (**c**) and (**d**) HVRs were extracted using in silio PCR with mothur (**b**) and (**d**) and HVR extraction with V-Xtractor (**a**) and (**b**) from sequences present in DAIRYdb v1.1 to test completeness of the sequences therein. While almost 100% of the 10’290 sequences span over V2 to V8 only 89% contain V1 and 68% contain V9 (**a**) and (**c**). The in silico PCR highlights the theoretical amplification efficiency of the most common universal primers with 0 mismatches normalized to the total number of detected HVR (**b**) and (**d**)

The single HVRs and HVR pairs were extracted from randomly subsampled sequences of the DAIRYdb using two methods: V-Xtractor [66] or in silico PCR with mothur [33] (Fig. 4a, c). While V-Xtractor extracted all present HVRs, the in silico PCR also evaluated the theoretical extraction efficiency of the different primer pairs (Table 1). Almost 100% of the sequences in the DAIRYdb span from V2 to V8. The HVR V1 (89%) and V9 (68%) are the regions with the lowest coverage in the DAIRYdb. This is due to the commonly used universal primers 8F and 1492R for the full-length 16S PCR leading to the entire or partial loss of V1 and V9 (Fig. 4a, Table 1). The primer pairs targeting V1 (25%), V2 (58%) and V9 (55%) were less efficient as compared to the primer pairs targeting V3 (84%) to V8 (77%). The primer pair for V4 performed best with 92% coverage, followed by V7 (88%), V5 and V6 (86%). The same hold true for the HVR pairs, where the HVR pairs V1V2 (38%), V2V3 (55%) and V8V9 (51%) performed worse as compared to the central HVR pairs (79–89%) (Fig. 4c).

**Table 1:** Primers used in the in silico PCR extraction of the HVRs

Label	Name - ARB primers	HVR	Location*	bp	Primer sequence	GC%	Reference	Original reference primer
8F_v1f	S-D-Bact-0008-d-S-20	v1F	8-27	20	AGAGTTTGATCMTGGCTCAG	50	[67]	
120R_v1r	*S-D-Bact-0120-e-A-20*	v1R	101-120	20	TTACTCACCCGTNCGCCRCT	55	mod. rev-compl. after [68]	
101F_v2f	S-D-Bact-0101-a-S-20	v2F	101-120	20	AGYGGCGNACGGGTGAGTAA	55	mod. after [68]	
355R_v2r	*S-D-Bact-0355-a-A-18*	v2R	338-355	18	GCWGCCTCCCGTAGGAGT	66	mod. after [69]	
338F_v3f	*S-D-Bact-0338-a-S-20*	v3F	337-354	20	ACWCCTACGGGWGGCAGCAG	65	mod. after [70]	
534R_v3r	S-D-Bact-0518-b-A-17	v3R	518-534	17	ATTACCGCGGCTGCTGG	65	[71]	
515F_v4f	S-*-Univ-0515-b-S-19	v4F	515-533	19	GTGNCAGCMGCCGCGGTAA	63	mod. after [72]	
806R_v4r	*S-D-Bact-0756-a-A-20*	v4R	787-806	20	GGACTACHVGGGTWTCTAAT	40	mod. after [72]	
784F_v5f	S-*-Univ-0779-a-S-15	v5F	784-798	15	RGGATTAGATACCCY	40	mod. after [73]	
926R_v5r	*S-D-Bact-0907-b-A-20*	v5R	907-926	20	CCGTCAATTYYTTTRAGTTT	25	mod. after [74]	
907F_v6f	S-D-Bact-0907-a-S-20	v6F	907-926	20	AAACTYAAARRAATTGACGG	25	[75]	
1114R_v6r	S-D-Bact-1114-b-A-16	v6R	1099-1114	16	GGGTYKCGCTCGTTRY	50	mod. after [73]	S-D-Bact-1114-a-A-16
1099F_v7f	S-*-Univ-1099-a-S-16	v7F	1099-1114	16	RYAACGAGCGMRACCC	50	new primer	S-*-Univ-1100-a-S-15
1200R_v7r	*S-D-Bact-1200-a-A-16*	v7R	1185-1200	16	GAYTTGACRTCVTCCM	38	new primer	
1185F_v8f	*S-D-Bact-1185-a-S-16*	v8F	1185-1200	16	KGGABCACCGCYCGYC	63	new primer	
1407R_v8r	*S-D-Bact-1407-a-A-16*	v8R	1391-1407	16	GRCGRGCGGTGWGTRC	63	mod. after [76]	S-D-Bact-1391-a-A-17
1391F_v9f	*S-D-Bact-1391-a-S-16*	v9F	1391-1407	16	GYACWCACCGCYCGYC	63	new primer	
1510R_v9r	S-*-Univ-1510-b-A-19	v9R	1492-1510	19	GGNTACCTTGTTACGACTT	42	mod. after [77]	S-*-Univ-1492-a-A-21

*E. coli position as a reference

The ratio between the number of detected HVRs with V-Xtractor and HVRs extracted by in silico PCR determined the biodiversity coverage of the different HVRs achieved with the different primer pairs, which can bias further downstream analyses depending on the HVR targeted (Fig. 4b, d). The net in silico performance of each primer pair is presented as normalized to the total number of sequences detected by V-Xtractor for each HVR (Fig. 4b, d).

The results of microbiome studies are more strongly influenced by the selection of the primer pairs and thereof of the HVR amplified, than by the sequencing technology used for the study [78–80]. The OTU-picking algorithm is mainly dependent on the sequencing technology (clustering vs. denoising) or ASVs instead [81]. Therefore, classification predictors are of secondary importance, although their impact on the outcome is not negligible [82]. The selection of the primer pairs should be made after careful consideration of their coverage in diversity with respect to the studied environment [82]. Although researchers tend to use primers as universal as possible to catch the entire diversity present in the samples, it might be a pragmatic approach to lose some universality while increasing specificity for the studied environment. For dairy products, the DAIRYdb achieves both, covering all the biodiversity expected in these environments, while achieving specificity in taxonomic annotation.

Annotation accuracy of the DAIRYdb was compared with other universal databases, such as Silva128, RDP trainset v16, LTP and Greengenes analysing fragments of single HVRs or HVR pairs extracted from the sequences in the DAIRYdb with V-Xtractor and in silico PCR. About 1000 subsamples, each composed of synthetic HVRs extracted from 100 randomly selected sequences from the DAIRYdb, by either V-Xtractor or in silico PCR, were assigned to all taxonomic ranks by the means of three different classification predictors (Blast+, Metaxa2 and SINTAX) and the aforementioned databases.

**Fig. 5:**
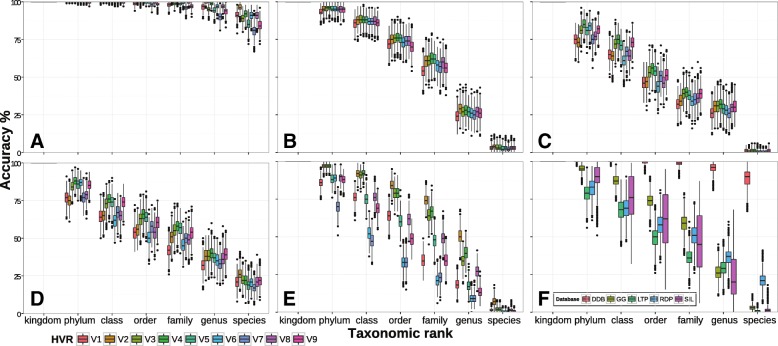
Taxonomy annotation accuracy of the DAIRYdb on reads extracted with V-Xtractor. Single HVR V1-V9 were re-annotated using three different classification algorithms, Blast+, Metaxa2 or SINTAX, respectively. This figure shows the results with SINTAX (Analyses with Metaxa2 and Blast+ are shown in Additional file 2). Taxonomy annotation was bootstrapped 1000 times with a subset of 100 randomly selected sequences from the DAIRYdb and annotated with DAIRYdb (DDB) (**a**), Greengenes (**b**), LTP (**c**), RDP (**d**) and Silva (**e**). Average performance of all HVR for each database (**f**) (accuracy = correctly annotated/total)

Single HVRs extracted with V-Xtractor and annotated with the DAIRYdb using SINTAX achieved annotation accuracy above 75% at all taxonomic ranks (Fig. 5a). Accuracy was highest for the even HVRs (V2, V4, V6 and V8) as compared to the odd HVRs (V1, V3, V5, V7 and V9). The region V2 presented the highest taxonomy annotation accuracy, which is in line with other findings showing that the regions V1 and V2 resulted in a more accurate OTU clustering at 97%, 98% and 99% [27]. Overall, the universal databases were less accurate with decreasing taxonomic rank (Fig. 5b-e). Only the RDP trainset v16 achieved about 25% of correct species annotations, while the other databases only classified to genus rank. Although the RDP trainset v16 performed best among all universal databases, annotation accuracy was below the accuracy values assessed in previous studies [48]. Universal databases achieved highest annotation accuracy with V4, with exception to Silva, which performed best on V2 (Fig. 5). Generally, the difference in annotation accuracy was stable through all HVRs with exception to the Silva database, where bigger oscillations were observed between the HVRs showing a clear drop for V6 and V7 (Fig. 5e). All HVRs taken together, the DAIRYdb achieved a significantly better taxonomy annotation accuracy (adjusted p-value <0.001) of average 88.9*%*±5.5 as compared to the universal databases tested at any taxonomic rank, but particularly at order to species ranks (Fig. 5f; Additional file 4: Tables S1 and S2). Results with Blast+ and Metaxa2 on single HVRs are available in Additional file 3.

**Fig. 6:**
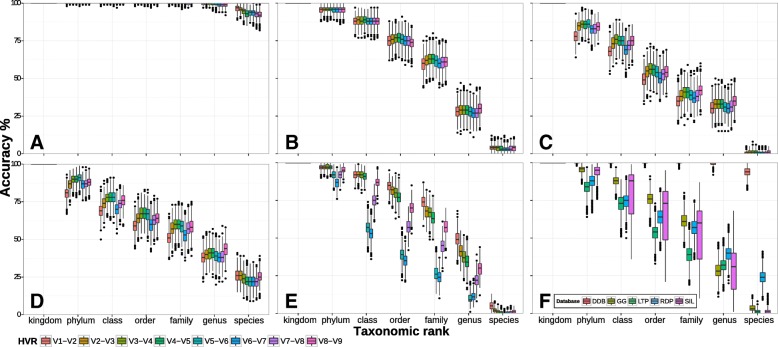
Taxonomy annotation accuracy of the DAIRYdb on reads extracted with V-Xtractor. The HVR pairs V1-V2, V2-V3, V3-V4, V4-V5, V5-V6, V6-V7, V7-V8, V8-V9 were re-annotated using three different classification algorithms, Blast+, Metaxa2 or SINTAX, respectively. This figure shows the results with SINTAX (Analyses with Metaxa2 and Blast+ are shown in Additional file 2). Taxonomy annotation was bootstrapped 1000 times with a subset of 100 randomly selected sequences from the DAIRYdb and annotated with DAIRYdb (DDB) (**a**), Greengenes (**b**), LTP (**c**), RDP (**d**) and Silva (**e**). Average performance of all HVR for each database (**f**) (accuracy = correctly annotated/total)

The results with the HVR pairs was similar to the single HVRs (Fig. 6a). Annotation accuracy between HVR pairs was less variable between different HVR pairs and within the bootstrapping values of the same HVR pair as compared to the single HVRs, indication for a more robust classification with increasing number of HVRs. The HVR pair V1V2 achieved the highest annotation accuracy at species rank in the DAIRYdb, as well as with RDP and Silva (Fig. 6d-e). These results are in agreement with previous studies, where V1 and V2 have been shown to have the highest average classification accuracy and average confidence estimate up to the genus rank [19]. Greengenes species annotation accuracy was similar for all HVRs, while LTP showed very low performance at species rank (Fig. 6b-c). The average accuracy value for correct species annotation of all HVR pairs with the DAIRYdb was over 94*%*±2.8 (Fig. 6f). Only species annotation with the RDP trainset v16 achieved 25% of correct annotations. The BLAST16S database was shown to obtain genus accuracy values ∼50% for V4, which improved with increasing length to ∼60% with V3–V5 and ∼70% with full-length 16S [52]. As expected, the increasing number of HVR increases the confidence in taxonomy annotation. Results with Blast+ and Metaxa2 on HVR pairs are available in Additional file 3.

**Fig. 7:**
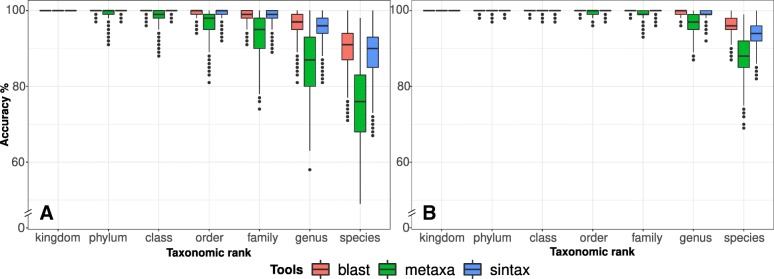
Comparison of the overall annotation accuracy of the three algorithms, Blast+, Metaxa2 and SINTAX for all single HVR (**a**) and HVR pairs (**b**). Although Blast+ presented a slightly better performance over SINTAX and Metaxa2, it was not statistically significant. All three classification tools assigned more than 75% of the sequences using the DAIRYdb as a reference for all HVR pairs

Different taxonomy predictors using the DAIRYdb showed similar performances and only little difference between single HVRs (Additional file 3: Figures S11) or HVR pairs (Additional file 3: Figure S12). Annotation accuracy performance varied more between different databases than between different classification predictors. The results confirm that OTUs annotation is primarily influenced by the selection of the database, by the HVR, and only at last by the taxonomy predictor (Additional file 3: Figures S1-S10). A comparison of the three classification predictors, Blast+, Metaxa2 and SINTAX with the DAIRYdb confirmed that HVR pairs could be more accurately assigned to the correct species than single HVR (Fig. 7). Among all tools, Blast+ and SINTAX were slightly better than Metaxa2. Since Metaxa2 uses more stringent parameters, as it only assigns the taxa if in agreement with Blast+, the lower performance of Metaxa2 with respect to Blast+ alone is therefore not surprising. Moreover, Metaxa2 performance is strongly dependent on the ANI thresholds used, which were set according to previous studies [83]. On the other hand, the more stringent parameters of Metaxa2 reduced the number of over-classified sequences. Generally, taxonomy annotation results are most robust whilst using different classification predictors with the DAIRYdb. We therefore recommend to use both, Metaxa2 with integrated Blast+ and SINTAX to obtain taxonomy annotations closest to the ground truth. Although a lower SINTAX cutoff of 0.6 increases the risk of over-classification, it is justified by the better quality of the DAIRYdb and the comparison with Metaxa2 for definitive taxonomy annotation (more details on the recommended usage on real samples are described on https://github.com/marcomeola/DAIRYdb).

**Fig. 8:**
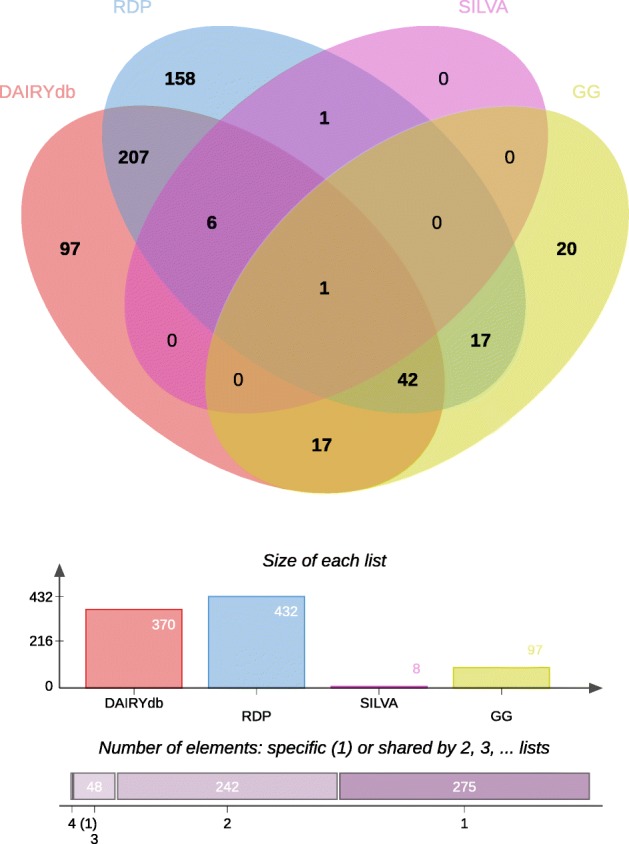
Taxonomy annotation accuracy test on sequences from the HITdb. Comparison of the taxonomy annotation accuracy at species rank between the DAIRYdb, RDP, Silva and Greengenes on type strain sequences present in the HITdb [50]. On sequences from origin other than dairy products, the DAIRYdb performs better than Silva and Greengenes, but not better than the RPD trainset v16

The main scope of the DAIRYdb is to improve accurate species classification in dairy products. Beyond this, it covers a considerable diversity in agreement with the diversity detected in dairy products so far. However, the DAIRYdb does not necessarily perform better than universal databases on a set of sequences from environments other than dairy, such as the human gut. Annotation accuracy performed on sequences from type strains included in the Human Intestinal Tract database (HITdb) showed that the DAIRYdb performed comparably well to the RDP trainset v16 and better than Silva and Greengenes (Fig. 8) [50].

The study of every particular environment calls upon peculiar requirements. Dairy products are no exception, as their bacterial communities are usually dominated by few phylogenetically highly related species, which are often difficult to discern, such as *L. casei*, *L. paracasei* and *L. rhamnosus* or *S. thermophilus* and *S. salivarius*. Particularly for *S. thermophilus*, which is a very important representative bacterium in dairy products, the official name still is *S. salivarius* subsp. *thermophilus* [84]. Although a separate full species status was proposed [85], persistent contention prevented a full ratification by the taxonomic committees [84]. The advances of genomics in microbiology has led to a reassessment of the phylogeny, which still remains a moving target particularly for microbial taxonomy [48, 60].

The correct description of a bacterial community structure remains a challenge in microbiome studies. Any parameter, from wet-lab (*i.e.*, DNA extraction, primer and HVR selection, amplification, sequencing) to the bioinformatic pipeline, can influence the outcome. Although the achievement of over 90% accurate species annotation of short 16S fragments can be considered a dramatic improvement, quality of dairy products is often influenced by different strains of the same species [86]. The resolution at strain or subspecies rank, however, based on full 16S is highly unlikely to be achieved independently from advancing sequencing technology. While on the one hand the definition of strains and subspecies is even more problematic than higher ranks such as species [87], on the other hand, the intraspecies variability of the 16S lacks sufficient resolution to clearly discern between strains and subspecies within the same species [88]. Nevertheless, recent powerful bioinformatics tools, such as Oligotyping [89], Minimal Entropy Decomposition (MED) [90], Dada2 [91] and DiTaxa [92], can be applied to distinguish between ecologically relevant ASVs. The resulting oligotypes or haplotypes within a species might be linked to different metabolic pathways or associated to identified physico-chemical characteristics of cheese or dairy products. Hereof, the DAIRYdb is a powerful improvement as it accurately identifies the sequences belonging to the same species, which can further be decomposed to oligotypes. Finally, links between oligotypes and 16S from WGS could improve the link between phylogeny and ecotypes for a better ecological understanding of the system [81, 87, 93].

Yet, the way and ability to recognize the basic unit for taxonomy of prokaryotes depends on the resolution power of the observational methods available [94]. Increasing sequence read lengths will make it possible to cover three HVRs or even the full 16S, thus further improving taxonomic annotation accuracy at species rank by using a manually curated databases like the DAIRYdb.

## Conclusions

Accurate prediction of taxonomy based on the marker gene 16S is a fundamental step in microbial diagnostics and microbial ecology studies. Dairy products, particularly cheeses, are enriched by a few dominant species often belonging to the same genera, such as *Lactobacillus* spp., *Lactococcus* spp., *Streptococcus* spp. An automatic and reliable taxonomic annotation to the correct species is pivotal to further routine microbial diagnostics.

Different to available universal databases, DAIRYdb achieved correct taxonomy annotation for ∼90% of species names on single HVRs and HVR pairs with 16S sequences present in dairy samples [52]. The better performance of the DAIRYdb over universal databases can be explained by the overall reduced number of sequences, only 10’290, with no conflicting taxonomy at all taxonomic ranks. Our results are in disagreement to the recommendation to use the largest and most diverse database possible for 16S classification [95]. On the opposite, manually curated 16S databases with authoritative full-length 16S sequences dedicated to the studied environment enormously improve classification confidence to the species rank [48–50]. Reducing the number of representative sequences to a minimal number in the training set further diminishes the risk of highly similar sequences with conflicting taxonomy, thus lowering the performance of the database used for classification [48, 49].

We therefore propose the manually curated DAIRYdb as a reference database for 16S microbiome studies on cheese and dairy products. The implementation of a curated database may lead to wider consensus and standardization processes reducing conflicts in literature due to the use of different universal databases integrated in different classification tools [82, 96].

## Methods

### DAIRYdb construction

To retrieve a comprehensive set of near full-length 16S sequences originating from cheese and dairy products, a search was performed against the NCBI Genebank nucleotide database using the command “CHEESE[All Fields] OR MILK[All Fields] OR TEAT[All Fields] OR STARTER[All Fields] OR WHEY[All Fields] OR DAIRY[All Fields] AND “16S ribosomal RNA”[All Fields] AND (“bacteria”[porgn] OR “archaea”[porgn]) AND 1000:2000[SLEN] NOT shotgun”, and the EMBL databases using the command “16S ribosomal RNA cheese, 16S ribosomal RNA milk, 16S ribosomal RNA starter, 16S ribosomal RNA teat, 16S ribosomal RNA dairy, 16S ribosomal RNA whey”. About 12’930 16S sequences were retrieved from NCBI and 51’175 from EMBL.

Mostly unpublished 16S sequences from INRA (171’282), as well as some recently published [97] (NCBI Bioproject PRJNA421256), and Agroscope (1’559) were also included after clustering at 99% (see below for details). The resulting set of 236’946 sequences constituted the starting pool for building up DAIRYdb. The extracted sequences of the starting pool were matched against the Greengenes v13_5, SSURef_NR99_123.1_tax_silva_trunc (Silva123) and RDP current_bacteria, RDP trainset v16_022016 16S databases using the *usearch_global* command of vsearch (v1.11.1) with parameters -id 0.8 and -mid 97. About 34’515 sequences were extracted from Greengenes, 29’181 from Silva123 and 52’686 from RDP, representing all possible full-length 16S sequences associated to the studied environment from the universal repositories prior to OTU clustering.

Altogether, the keywords associated sequences (236’946) and their representative database sequences (116’382) amounted to a total of 391’672 16S sequences of different length and quality. The extracted sequences were first dereplicated with priority to full-length sequences (279’737) and minimal length of 300 bp (268’613) if no full-length was present using the *derep_fulllength* and *derep_prefix* of usearch (v6.0.307_i86osx32), respectively.The remaining 268’613 sequences were divided into “good” sequences, *i.e.,* >1300 bp (50’309), and “bad” sequences, *i.e.,* <1300 bp (218’304), using Prinseq (v0.20.4). The “bad” sequences were matched against all databases (Greengenes, Silva and RDP) to obtain reference sequences of better quality and length using the *usearch_global* command of vsearch (v1.11.1) with parameters -id 0.5 and –maxhits 1. The 146’355 hit sequences were dereplicated using the *derep_fulllength* and *derep_prefix* of usearch6.0.307_i86osx32 resulting in 92’722 unique 16S sequences that were merged with the initial 50’309 “good” sequences. To remove redundancy, the 143’031 16S sequences were dereplicated again, reducing the total amount to 98’590 sequences and subsequently divided based on the length threshold of 1300 bp using Prinseq (v0.20.4) for clustering purposes. The sequences were first subjected to chimeric sequence removal using the 16S reference database available at http://www.drive5.com/uchime/rdp_gold.fa with usearch (v6.0.307_i86osx32) and subsequently clustered to OTUs separately to a similarity threshold of 97% in steps of 0.5% using the *cluster_otus* command in usearch (v6.0.307_i86osx32). The resulting 18’457 OTUs were matched against a database composed of full-length 16S sequences of type strains from Silva126 using the parameters –id 0.5 –mid 97 -maxhits 1. The sequences with no match were matched again, however, using less stringent parameter –id 0.5 -maxhits 1. Finally, the resulting 14’468 sequences were dereplicated, clustered at 97% and cleaned by removing eukaryotic, chloroplastic and mitochondrial sequences, Pintail value <75 and <1200 bp. The intermediate version of the DAIRYdb contained 9’739 representative 16S sequences.

Species whose sequences were present before the clustering at 97% and absent after clustering have been reintroduced if identical sequence with synonymous taxonomic identity was absent in the DAIRYdb (see List of Prokaryotic Names validly published of the Deutsche Sammlung von Mikroorganismen und Zellkulturen (DSMZ)). Moreover, 142 missing species listed in [56] and [57] were added to the DAIRYdb.

### DAIRYdb curation

The taxonomy of the 9’963 16S sequences assigned by Silva126 was checked manually upon consistency between the six top taxonomic ranks (Kingdom to Genus). The taxonomy annotations of these sequences were mostly obtained from authoritatively named type strains. While there could be some errors in the taxonomy annotations in Silva, or discordance to DSMZ, taxonomy annotations from authoritative type strains were considered as truth standards for the SATIVA test and manual curation. After a first manual curation, three closest type strain sequences with an authoritative taxonomy [48] were retrieved for all representative sequences in the DAIRYdb from Silva126 at 0.99 ANI and RDP (SequenceMatch: “typestrains”, “isolates”, “good quality”, >1200 bp). The 7’244 closest neighbours (CN) were dereplicated and the resulting 4’545 CN type strain sequences were added to the DAIRYdb. The CN were used as references in the curation process with the Semi-Automatic Taxonomy Improvement and Validation Algorithm (SATIVA [59]) called with the command “sativa.py -s input.phyl -t input_taxonomy.txt -x BAC -T 36 -N 20 -S” using the authoritative CN type strain sequences as reference after alignment with SINA (v1.2.11 [58]).

Manual curation of the taxonomy was performed based on both, SATIVA results and RAxML tree with the sequences in the DAIRYdb and the authoritative CN [98] using the command “raxmplHPC-PTHREADS-AVX2 -T 36 -f a -k -x 237 -m GTRGAMMA -p 1481544944 -N autoMRE -s input.phyl -n output.tre -O -w”. Using the algorithms Metaxa2 and SINTAX, an additional training set of Silva126 type strains was used to obtain a taxonomic proposition for sequences with unclear taxonomy. This step was iterated several times until all possible sequences could be assigned to a species group. The sequences with no unequivocal annotation where identified with LPT followed by an underscore and respective taxonomic rank (*e.g.*, *XXX_Family*, *Lactobacillus_Species*).

At least one type strain for each bacterial species present in the DAIRYdb was added if available in the DSMZ database bacdive.dsmz.de/ (https://bacdive.dsmz.de/). After manual curation the 4’545 CN reference sequences with authoritative taxonomy were removed. The final DAIRYdb.v1.1 contains a total of 10’290 16S sequences.

### DAIRYdb validation and statistical analyses

Single HVR and pairs of HVR were extracted in silico from the 10’290 16S sequences of the DAIRYdb using primers listed in Table 1 with the *pcr.seqs* function from mothur with 0 missmatches. All present single and pairs of HVRs in the 16S sequences were extracted with V-Xtractor, which is based on a HMM algorithm for the HVR detection and where extraction occurs based on the presence of the HVR with no primer biases.

Taxonomy annotation accuracy was assessed with 1000 subsamples of each 100 randomly selected sequences from within the DAIRYdb. Annotation accuracy is defined as the fraction of sequences that are correctly predicted and classified at each rank [52]. The sequences were taxonomically assigned at all taxonomic ranks by means of three classification tools (Blast+, Metaxa2 and, SINTAX). Annotation accuracy was assessed using 4 universal databases: Greengenesv13.5 (1’262’986 sequences) [45], SILVAv128SSURefNr99 (645’151 sequences) [40], LTPv123SSU (11’939 sequences) [42], RDPtrainingsetv16 (13’212 sequences) [43] and the DAIRYdb (10’290 sequences). Correct annotation at all taxonomic ranks was assessed comparing the resulting assignment with the reference DAIRYdb. Pairwise comparisons using Wilcoxon rank sum test were used to highlight significant differences between database assignment accuracy. The *p-values* obtained were adjusted using Bonferroni correction. For more details on the statistical evaluation process see Additional file 4.

### Visualizations

#### Krona

A Krona chart was generated using KronaTools [99]. The total sum of each unique taxonomy of the DAIRYdb was assessed and used as input in the ktImportText command.

#### Venn diagram

A Venn diagram to determine the potential origin of each sequence composing the DAIRYdb was generated with the web app Jvenn [100]. The global alignment -usearch_global module of vsearch to per a global alignment between the 10’290 sequences of the DAIRYdb and each raw database corresponding to keywords: dairy, cheese, starter, whey, milk. These raw databases were previously dereplicated. We used the parameter -strand both -id 0.9 -maxaccepts 100 -maxrejects 100. For each keywords, the matching sequence identifiers were used as input in Jvenn to generate the Venn diagram.

## Additional files

Additional file 1 Species list. The additional file 1 is a text file with all species included in the DAIRYdb in alphabetical order. Can be opened with any text editor or spreadsheet software. (TXT 100 kb)

Additional file 2 Krona diagram. Additional file 2 is an html file with a Krona diagram showing the complete diversity present in the DAIRYdb can be interactively inspected in a webbrowser. (HTML 872 kb)

Additional file 3 Supplementary Information. Additional file 3 contains supplementary figures described in the main manuscript. It is a portable document file (pdf) that can be read with Acrobat Reader. (PDF 3925 kb)

Additional file 4 RMarkdown for reproducibility. The additional file 4 includes the different scripts used to test all HVR primers (for single HVR and HVR pairs), scripts used to customize the different database in order to use them in the different assignation tools, and scripts used for DAIRYdb validation. We mainly used bash and R scripts [101, 102]. The file is in html format and was generated starting from a Rmarkdown file. (HTML 1012 kb)

## Data Availability

The DAIRYdb is available on a public repository (https://github.com/marcomeola/DAIRYdb). The different databases used to validate the DAIRYdb are available on their own website.

## Abbreviations

ANI: Average nucleotide identity
ASV: Amplicon sequence variant
BacDive: Bacterial diversity metadatabase
CN: Closest neighbour
DAIRYdb: Dairy agroscope INRA ribosomal accuracY database
DGGE: Denaturing gradient gel electrophoresis
DNA: Deoxyribonucleic acid
DSMZ: Deutsche Sammlung von Mikroorganismen und Zellkulturen
GG: Greengenes database
HITdb: Human intestinal tract database
HMM: Hidden Markov-Monte-Carlo model
HVR: Hyper variable region (16S)
INSDC: International nucleotide sequence database collaboration
LAB: Lactic acid bacteria
LCA: Lowest common ancestor
LCR: Lowest common rank
LH-PCR: Length heterogeneity polymerase chain reaction
LPSN: List of prokaryotic names with standing in nomenclature
LSU: Large subunit
LTP: Living tree project
MED: Minimal entropy decomposition
NCBI: National center for biotechnology information
NGS: Next generation sequencing
OTU: Operational taxonomic unit
RDP: Ribosomal database project
rRNA: Ribosomal ribonucleic acid
SSU: Small subunit
T-RFLP: Terminal restriction fragment length polymorphism
WGS: Whole genome shotgun
