# Supplementary material for: DAIRYdb: a manually curated reference database for improved taxonomy annotation of 16S rRNA gene sequences from dairy products
Source: BMC Genomics. 2019 Jul 8;20:560. doi: 10.1186/s12864-019-5914-8 (PMC6615214; doi:10.1186/s12864-019-5914-8)
Supplement: Supplementary file 2 — Krona diagram. Additional file 2 is an html file with a Krona diagram showing the complete diversity present in the DAIRYdb can be interactively inspected in a webbrowser. (HTML 872 kb) [file 12864_2019_5914_MOESM2_ESM.html]

Javascript must be enabled to view this page.

magnitude
magnitudeUnassigned

final\_counts2

10290

107

79

5

5

5

2

2

1

1

1

1

1

1

29

3

3

3

2

1

11

3

3

1

1

1

8

1

1

1

1

3

3

1

1

1

1

1

1

15

1

1

1

4

1

1

2

2

1

1

4

4

2

2

6

2

1

1

1

1

1

1

2

1

1

2

2

1

1

1

1

1

1

24

23

1

1

1

1

1

1

21

3

1

1

1

9

2

1

1

1

2

1

1

9

1

1

1

1

1

1

1

1

1

1

1

1

1

19

19

4

4

4

15

15

15

10

1

1

1

1

1

2

2

2

2

2

1

1

1

1

1

1

1

1

1

1

1

1

1

1

1

3

1

1

1

1

2

2

1

1

1

1

1

1

1

1

1

14

14

9

1

1

1

8

8

8

3

3

1

1

1

1

1

1

2

1

1

1

1

1

1

3

3

3

3

3

1

2

1

1

1

1

1

1

10183

11

11

11

11

11

11

249

43

43

43

37

37

6

6

33

33

21

9

9

11

11

1

1

12

12

12

2

2

2

2

2

98

98

6

6

6

92

2

1

1

3

3

56

56

2

1

1

1

1

1

1

27

27

1

1

1

1

1

72

8

8

8

8

1

1

1

1

63

54

1

1

9

9

38

38

6

6

9

9

9

1

1

1

1

1

1

20

20

20

20

20

20

3832

760

337

128

9

1

2

1

2

1

1

1

119

1

1

1

4

1

1

1

1

1

1

1

1

1

1

1

1

1

1

1

1

1

1

2

1

1

1

1

1

1

1

1

1

1

1

1

1

1

1

1

1

1

1

1

1

2

1

1

1

1

1

1

1

1

1

1

1

1

1

1

1

1

1

1

1

1

1

1

1

1

1

1

4

1

1

2

1

1

1

1

1

2

1

1

1

1

1

1

1

1

2

1

1

1

1

1

1

1

1

3

1

1

1

1

1

1

1

67

10

1

2

1

1

1

2

1

1

57

1

1

1

1

1

2

3

2

2

1

1

2

1

1

1

1

1

2

1

1

1

1

1

2

1

1

1

1

1

1

3

1

2

1

1

1

2

4

1

1

1

1

41

1

1

3

2

1

2

1

1

1

1

1

1

2

1

1

1

1

2

2

10

10

6

1

1

1

3

8

1

1

1

2

1

2

1

1

1

1

2

1

1

46

7

1

1

1

1

2

1

1

1

2

2

2

1

1

2

2

1

1

31

1

1

1

2

3

1

1

2

1

1

1

1

1

1

1

1

1

3

1

1

1

1

1

2

30

2

2

1

1

1

1

1

1

1

1

2

1

1

13

4

3

2

1

1

1

1

1

1

8

1

2

1

1

1

1

1

25

1

1

11

1

1

1

1

1

1

1

1

1

1

1

10

1

1

1

1

1

1

1

3

3

1

1

1

1

1

1

1

422

3

3

1

1

1

6

5

2

1

1

1

1

1

25

4

1

1

1

1

1

1

4

1

2

1

1

1

1

1

1

1

1

1

1

1

2

1

1

2

1

1

2

1

1

1

1

1

1

1

1

2

2

111

10

1

1

1

1

1

1

1

2

1

81

1

1

1

1

1

1

1

1

2

1

1

2

1

2

1

1

1

1

1

1

1

2

1

1

1

2

1

1

2

1

1

1

1

1

1

3

1

1

1

1

1

1

1

2

2

1

1

1

1

1

1

1

1

1

1

1

3

1

2

1

1

1

1

1

2

1

1

1

1

4

1

1

1

1

1

1

5

2

1

2

1

1

8

1

1

1

1

1

1

2

1

1

1

161

9

1

1

1

1

1

3

1

5

2

1

1

1

1

1

2

1

1

3

1

1

1

2

1

1

1

1

2

1

1

3

3

1

1

2

2

1

1

1

1

1

1

1

1

3

1

2

2

1

1

102

2

10

1

2

1

1

1

2

1

1

1

3

1

1

1

1

1

1

1

1

1

1

1

1

1

1

1

1

1

1

1

1

1

1

2

1

1

1

1

1

1

1

1

1

3

2

1

1

1

1

3

1

1

1

1

1

2

2

1

1

2

1

1

2

1

1

1

1

1

1

2

1

1

2

1

1

1

1

1

1

1

1

1

1

1

1

1

1

3

1

2

2

1

1

1

1

1

1

1

1

1

1

1

1

1

1

1

1

1

1

6

6

1

1

1

1

1

1

8

7

1

1

1

1

1

1

1

1

1

31

1

1

1

1

1

1

1

1

1

1

4

1

1

1

1

9

1

1

1

1

1

1

1

2

2

1

1

1

1

1

1

8

1

2

1

2

2

1

1

16

12

2

2

1

1

1

1

1

2

1

4

3

1

1

1

1

51

2

2

8

1

1

1

1

2

2

3

1

1

1

4

2

1

1

1

1

33

1

1

2

1

1

1

1

1

1

1

1

1

1

1

1

1

1

1

1

1

1

2

1

1

1

1

1

1

1

1

1

2

1

1

1

1

2771

1

1

1

1

2726

8

8

8

1

1

1

86

3

1

1

1

1

1

20

1

1

1

2

2

1

1

1

1

1

1

3

1

1

1

1

2

1

1

7

7

3

3

16

16

6

1

1

1

1

2

5

1

1

1

1

1

3

3

3

1

2

2

2

13

10

1

1

1

2

2

22

22

12

7

3

1

1

1

31

11

6

2

3

7

4

2

1

3

1

2

5

2

3

1

1

1

1

3

3

9

3

3

6

2

4

81

81

81

978

11

3

8

7

5

2

6

1

5

9

9

13

13

60

58

2

36

36

2

1

1

39

10

7

7

2

13

1

1

129

129

29

26

2

1

2

2

14

4

10

354

354

129

1

3

2

1

1

1

103

1

1

1

7

1

1

5

8

7

1

3

3

7

4

3

1

1

48

46

2

2

1

1

47

1

13

33

14

14

7

7

4

4

1

1

2

219

25

25

193

193

1

1

24

6

6

11

1

2

1

1

1

2

3

5

5

1

1

1

1

780

5

5

33

3

1

1

1

1

3

16

1

2

1

2

1

9

7

1

1

3

3

188

186

2

4

2

2

33

33

13

2

8

3

8

6

1

1

25

23

2

3

3

10

5

1

4

1

1

2

1

1

1

1

1

1

1

1

26

26

34

1

15

1

5

1

11

2

2

1

1

1

1

7

7

11

11

1

1

12

12

2

2

5

5

12

2

10

8

2

6

1

1

9

1

8

1

1

13

2

3

1

7

18

18

6

6

74

3

1

1

1

1

2

1

1

1

1

6

1

4

10

1

1

14

1

3

1

5

1

2

3

1

1

4

2

41

41

3

3

2

1

1

1

1

14

14

5

3

2

4

2

2

5

4

1

3

3

3

2

1

5

1

1

1

2

4

1

1

2

8

8

12

12

1

1

12

11

1

5

2

3

5

1

1

2

1

5

5

10

1

1

3

1

4

5

5

2

1

1

6

3

3

8

8

2

2

15

1

1

7

2

3

1

3

3

7

7

9

3

2

1

5

5

1

1

1

1

1

72

7

6

1

14

3

2

1

3

2

1

2

21

1

1

1

1

2

3

1

1

1

1

1

1

2

4

12

12

1

1

11

3

2

1

5

4

4

2

2

152

1

1

1

1

6

4

2

1

1

2

2

1

1

2

2

1

1

74

1

1

2

1

1

1

1

1

2

1

1

1

2

1

3

1

1

1

1

1

2

2

1

1

1

3

1

1

1

1

1

1

1

2

1

1

1

1

4

1

2

2

1

1

1

1

1

1

4

1

1

1

1

1

1

1

4

4

1

1

1

1

1

1

1

1

13

1

3

1

1

7

5

1

1

1

1

1

11

10

1

7

1

1

1

1

1

1

1

13

1

5

5

1

1

1

1

2

2

3

3

16

1

1

1

1

1

1

1

1

12

12

51

13

5

2

2

4

35

35

3

3

102

34

33

1

19

1

17

1

1

1

22

22

26

1

1

18

5

1

8

1

1

7

6

1

32

2

2

3

3

3

1

1

1

2

2

8

8

2

2

5

5

1

1

6

1

1

1

3

1

1

1

1

1

1

37

6

6

2

1

1

1

1

4

4

4

4

3

1

2

1

1

2

2

1

1

1

1

5

1

1

1

1

1

2

1

1

5

1

1

1

2

5

5

5

5

25

1

1

1

1

1

1

1

1

1

2

2

2

20

2

1

1

4

4

1

1

3

1

1

1

1

1

1

1

1

1

1

1

3

3

1

1

1

1

1

1

1

1

1

1

1

1

1

1

11

11

7

4

2

1

1

1

3

1

1

1

1

1

1

1

4

4

4

4

4

8

8

8

8

8

1

1

1

1

1

1

1

1

1

1

187

187

187

4

4

2

1

1

55

55

12

4

1

4

3

1

1

23

1

1

1

10

10

1

1

1

1

2

2

4

4

5

5

3

3

1

1

1

1

5

5

32

31

1

25

25

5

5

1

1

4

4

1

1

1

1

1

4

4

4

4

4

1

1

1

1

1

1

1

1

1

1

93

93

15

5

5

1

1

2

1

1

7

2

5

78

1

1

2

1

1

15

1

3

4

2

1

2

1

1

1

1

9

1

1

5

1

1

3

3

2

2

1

1

1

1

6

1

1

3

1

2

2

1

1

1

1

1

1

2

1

1

4

1

1

1

1

6

1

1

1

1

2

1

1

1

1

2

2

1

1

1

1

4

1

2

1

10

8

2

9

9

9

9

9

9

1

1

1

1

1

1

83

83

57

57

57

1

1

55

26

26

26

26

1

1

1

1

1

1

101

30

30

30

30

30

23

23

23

1

1

2

2

9

1

1

3

4

1

1

5

5

5

1

1

3

35

35

25

25

25

10

6

3

3

4

4

1

1

1

1

1

12

7

7

7

7

5

5

2

2

1

1

2

2

1

1

1

1

1

1

83

8

8

8

8

8

36

2

2

2

1

1

17

17

1

1

1

1

1

1

1

1

1

1

1

1

1

1

2

1

1

2

1

1

2

2

1

1

1

1

1

1

1

1

7

7

1

1

2

2

2

2

1

1

1

1

10

10

1

1

1

1

1

1

1

1

1

1

2

2

2

1

1

1

1

39

34

34

34

34

4

4

4

4

1

1

1

1

19

19

19

4

4

4

2

2

2

6

6

6

1

1

1

3

3

3

3

3

3

14

14

12

7

7

1

1

1

1

2

1

5

5

5

2

2

1

1

1

1

10

10

10

10

10

10

130

130

130

3

2

1

1

1

1

4

4

4

123

5

5

8

8

1

1

66

4

1

1

4

1

1

1

3

3

1

2

1

1

1

1

19

2

1

1

1

1

1

1

2

1

2

1

1

2

4

1

1

11

2

1

2

6

3

3

28

2

1

18

1

1

1

1

1

1

1

4

4

4

4

4

4

30

1

1

1

1

1

22

22

19

8

8

11

11

3

3

3

7

7

7

7

7

16

3

3

3

3

3

12

12

1

1

1

8

8

7

1

3

3

3

1

1

1

1

1

920

638

8

8

5

1

3

1

2

1

1

1

1

26

26

1

1

1

1

2

1

1

22

1

1

1

1

1

1

1

1

1

1

4

2

1

1

1

1

1

1

93

49

1

1

2

1

1

3

3

39

1

1

2

2

2

1

1

1

4

1

1

2

2

1

1

1

1

2

1

1

4

1

2

3

3

1

2

1

1

44

1

1

4

3

1

2

2

3

2

1

10

1

2

1

1

1

1

1

1

1

6

2

1

2

1

5

5

1

1

4

1

1

1

1

1

1

1

1

1

1

2

1

1

2

2

1

1

45

9

9

2

6

1

4

1

1

2

1

1

1

1

6

6

1

1

2

2

10

1

1

3

3

6

4

2

7

7

7

9

7

2

1

4

1

1

1

1

31

31

9

2

1

1

2

1

1

1

1

1

6

1

1

1

2

1

1

1

1

1

1

1

1

1

3

1

1

1

3

1

1

1

1

1

1

1

1

1

1

1

1

1

191

5

5

5

3

1

1

2

2

50

4

1

1

1

1

3

2

1

2

2

3

2

1

2

2

1

1

1

1

11

1

1

1

1

1

1

1

1

1

1

1

23

1

1

1

1

3

1

2

1

1

1

1

3

1

1

1

1

1

1

2

2

1

1

14

1

1

4

4

1

1

3

1

1

1

1

1

1

1

3

1

1

1

2

1

1

1

1

8

3

2

1

2

1

1

3

1

2

6

6

1

4

1

18

2

2

1

1

15

2

1

1

1

1

1

1

1

2

1

1

1

1

6

3

1

1

1

2

1

1

1

1

3

1

1

2

1

1

14

1

1

12

1

1

1

1

1

1

1

1

1

1

1

1

1

1

5

2

1

1

1

1

1

1

1

1

2

1

1

1

1

48

2

1

1

1

1

14

2

1

1

1

1

1

1

1

1

1

1

1

1

1

1

3

2

1

1

1

1

1

1

1

1

1

9

1

1

2

1

2

2

1

1

1

1

1

1

5

2

1

1

1

1

1

1

1

1

1

3

1

2

1

1

1

4

4

1

1

1

1

23

1

1

1

1

1

1

9

1

1

2

1

1

6

1

2

1

1

1

6

1

1

1

1

1

1

1

1

2

1

1

6

3

1

1

1

1

1

2

1

1

2

2

2

1

1

17

17

15

1

1

1

1

1

1

1

1

1

1

1

2

1

1

1

1

1

1

96

23

10

1

1

2

1

1

1

1

1

1

2

1

1

7

1

1

1

1

1

2

4

1

1

1

1

10

10

1

1

1

2

1

1

1

1

1

55

54

1

1

1

1

1

1

1

1

1

1

1

1

2

2

1

1

1

1

2

1

1

1

1

1

1

1

1

1

1

1

1

1

1

1

1

1

1

1

1

1

1

1

1

1

1

1

1

1

1

1

1

1

1

2

2

1

1

5

4

1

1

1

1

1

1

1

1

1

67

4

1

1

3

2

1

50

1

1

1

1

5

1

3

1

3

1

2

2

2

2

1

1

30

1

2

1

1

1

3

1

1

1

1

2

1

1

1

2

1

1

1

1

1

1

1

2

1

1

1

5

3

1

1

1

1

1

4

4

4

8

6

2

1

1

1

1

2

2

26

26

2

2

1

1

4

1

3

1

1

7

2

5

1

1

1

1

1

1

3

1

2

1

1

3

1

2

1

1

2

2

2

1

1

11

11

11

11

94

94

6

4

3

1

2

2

4

4

4

84

12

9

3

67

31

36

1

1

4

4

101

101

101

6

1

1

1

1

1

1

10

2

2

5

1

1

1

52

52

1

1

1

1

1

1

4

4

1

1

1

1

5

2

1

1

1

1

1

13

1

6

3

1

1

1

4

2

2

3

3

3

3

1

2

74

1

1

1

1

39

1

1

1

2

2

2

31

31

31

5

5

5

34

34

34

34

4

4

4

4

1

2

1

6

2

2

2

2

4

4

4

4

6

6

6

6

1

1

1

1

1

1

1

1

1

1

1

1

41

25

25

25

25

25

16

16

16

3

1

2

1

1

12

2

8

2

24

24

24

17

17

17

2

1

1

1

1

5

5

5

29

28

28

27

17

1

15

1

10

8

1

1

1

1

1

1

1

1

1

1

3

3

3

3

3

3

86

1

1

1

1

1

3

3

3

3

3

75

75

75

75

75

2

2

2

2

2

1

1

1

1

1

4

4

4

4

4

112

4

4

4

4

4

108

108

108

108

108

77

61

1

1

1

1

59

57

2

2

1

1

3

3

3

3

10

10

2

2

7

4

1

1

1

2

1

1

6

6

2

1

1

8

8

2

2

8

8

1

1

2

2

2

1

1

1

1

1

1

1

1

1

6

6

6

6

6

9

3

3

3

3

6

6

6

6

34

34

34

22

3

2

1

5

1

2

2

1

1

1

1

12

2

3

1

2

2

1

1

12

1

1

2

2

1

1

8

1

1

3

1

2

13

1

1

1

1

1

12

12

1

1

1

11

1

1

4

4

6

1

1

4

3

3

3

3

1

1

2

1

1

256

1

1

1

1

1

1

1

1

1

1

33

5

5

5

5

23

23

23

23

5

5

1

1

3

1

2

1

1

25

11

4

4

1

3

7

7

7

13

13

13

13

1

1

1

1

10

10

10

10

10

33

33

33

14

1

13

1

1

18

18

2

2

2

2

2

4

4

4

4

4

3

3

3

3

3

8

8

8

8

8

14

5

5

5

5

3

3

1

1

2

1

1

6

6

6

1

5

12

12

12

12

12

7

7

7

7

7

1

1

1

1

1

26

26

26

26

26

2

2

2

2

2

56

56

56

21

20

1

9

9

4

4

1

1

5

5

1

1

5

5

7

7

3

3

17

17

2

2

2

3

3

3

12

12

12

1

1

1

1

1

24

24

24

24

2

1

1

3

3

1

1

3

3

1

1

2

2

3

3

2

2

1

1

2

2

4

3

1

3

3

3

3

3

3

3

2

2

2

2

2

1

1

1

1

1

2311

529

1

1

1

1

2

2

1

1

1

1

96

52

10

10

2

1

1

1

1

8

2

1

5

7

7

1

1

1

1

1

1

1

1

1

1

4

2

2

1

1

2

1

1

12

12

44

2

1

1

4

3

1

8

8

16

4

6

1

1

4

3

1

2

5

5

1

1

5

4

1

3

3

3

3

44

15

1

1

13

3

1

1

1

7

1

1

24

3

3

10

10

2

2

9

9

5

4

4

1

1

63

6

1

1

3

3

1

1

1

1

6

6

1

2

1

1

1

49

1

1

1

1

38

1

1

3

1

1

1

1

1

1

1

1

1

1

1

1

1

1

1

2

6

1

2

2

1

1

2

1

6

5

1

3

3

2

2

1

1

57

28

21

2

4

1

1

1

4

2

1

2

1

1

1

2

1

1

5

5

29

1

1

5

2

2

1

5

5

17

2

3

1

2

1

3

5

1

1

5

3

3

1

2

2

2

2

39

1

1

1

4

3

3

1

1

28

1

1

27

1

20

5

1

3

3

3

3

3

3

28

11

3

3

4

4

4

4

17

1

1

1

1

15

15

37

37

3

3

34

34

29

29

1

1

18

17

1

1

1

9

9

1

1

1

1

124

1

1

1

32

5

5

7

7

1

1

1

1

9

6

3

8

1

7

1

1

21

11

1

9

1

7

1

1

5

1

1

2

2

5

5

5

1

1

1

33

1

1

32

1

31

20

20

20

11

1

1

4

4

2

2

1

1

3

3

716

22

22

1

1

1

1

7

1

2

1

1

1

1

1

1

1

1

2

1

1

4

1

1

1

1

4

2

1

1

1

1

29

2

2

1

1

14

2

2

1

1

1

1

1

1

3

1

1

1

6

2

3

1

1

1

1

2

2

1

1

3

1

1

2

1

1

6

1

1

3

1

1

1

1

1

1

1

1

1

1

11

7

7

1

2

1

1

2

3

3

1

1

1

1

1

1

65

8

2

1

1

6

6

3

3

1

2

5

2

1

1

3

1

1

1

18

1

1

1

1

1

1

15

1

1

1

2

1

1

1

1

1

1

1

1

1

1

31

1

1

1

1

1

1

2

2

3

2

1

3

3

3

3

1

1

5

1

1

1

1

1

6

1

2

1

1

1

2

1

1

3

2

1

9

9

4

4

3

3

2

2

15

15

1

1

2

1

1

12

1

1

1

1

2

1

1

1

1

1

1

73

2

1

1

1

1

1

1

1

54

2

2

4

2

1

1

1

1

3

1

1

1

2

1

1

5

1

1

2

1

2

2

6

1

1

1

1

1

1

14

1

3

1

1

2

1

2

1

2

3

1

1

1

1

1

1

1

6

6

1

1

2

2

1

1

3

2

2

1

1

9

2

2

7

1

1

4

1

4

1

1

3

3

37

3

3

1

1

1

15

2

1

1

1

1

1

1

7

1

2

1

2

1

2

1

1

2

1

1

5

5

1

1

1

1

1

8

4

1

2

1

4

1

3

1

1

1

2

2

1

1

2

2

1

1

1

1

1

132

3

3

1

2

3

3

3

7

7

7

80

2

1

1

2

1

1

1

1

1

1

39

39

1

1

2

1

1

3

1

1

1

1

1

2

2

1

1

2

2

23

8

6

1

8

17

2

1

1

1

1

1

1

1

1

1

1

1

1

2

1

1

1

1

1

1

6

6

19

1

1

4

4

13

1

11

1

1

1

3

2

1

1

1

1

20

8

3

3

2

2

2

2

1

1

12

2

1

1

10

1

1

1

1

1

1

1

3

3

3

1

1

2

1

1

18

12

12

12

6

6

2

3

1

65

2

2

2

32

3

3

29

4

6

3

3

1

1

1

1

1

1

1

1

1

4

31

9

9

3

3

10

5

3

2

9

9

23

23

23

23

1

1

1

1

7

7

1

1

2

1

1

2

2

1

1

1

1

2

2

1

1

1

1

93

51

47

1

1

1

1

1

1

1

1

1

1

1

1

1

2

1

1

1

1

1

1

1

2

1

3

1

1

1

1

1

1

1

2

1

1

1

1

1

1

1

1

1

1

4

4

42

15

2

1

1

1

1

1

1

2

2

1

1

1

1

1

2

1

1

2

2

1

1

3

3

1

1

17

1

1

1

1

1

1

1

1

1

1

1

1

1

1

1

2

1

1

1

1

1

1

1

1

89

89

1

1

2

2

1

1

1

1

4

1

1

1

1

1

1

2

1

1

5

2

1

1

1

2

1

1

2

1

1

1

1

2

2

1

1

5

1

2

2

5

1

1

1

1

1

1

1

1

1

9

1

1

1

1

1

1

2

1

1

1

1

1

1

1

3

1

2

9

1

1

2

2

3

5

1

1

1

2

1

1

8

1

1

1

1

1

2

1

2

2

1

1

1

1

3

1

1

1

7

2

1

1

1

1

1

743

1

1

1

1

33

15

1

1

2

1

1

5

1

1

1

1

1

7

1

2

4

18

2

1

1

1

1

5

1

1

3

1

1

5

5

2

1

1

2

2

1

1

1

1

15

15

15

15

1

1

1

1

9

9

9

4

5

247

14

1

1

1

1

5

1

1

3

2

2

1

1

2

2

1

1

1

1

21

4

3

1

2

1

1

2

1

1

12

12

1

1

23

1

1

1

1

2

1

1

1

1

3

3

5

4

1

1

1

2

1

1

1

1

6

1

5

66

14

4

1

1

1

6

1

10

1

9

11

2

2

1

3

1

2

1

1

8

8

1

1

2

1

1

12

2

2

1

1

2

2

2

1

1

2

1

1

2

2

2

2

5

5

1

1

1

1

1

7

1

1

5

1

1

1

1

1

1

1

16

1

1

8

8

1

1

3

2

1

1

1

2

2

13

4

4

1

1

1

1

1

1

3

2

1

1

1

2

2

3

3

3

14

13

13

1

1

4

1

1

2

1

1

1

1

3

3

1

2

10

1

1

1

1

1

1

1

1

6

1

3

2

15

4

1

3

1

1

7

1

1

2

1

2

1

1

2

1

1

7

1

1

1

1

5

1

1

1

1

1

26

2

1

1

5

1

1

1

1

1

2

1

1

1

1

1

1

13

1

1

2

1

8

1

1

1

1

15

6

3

1

2

3

3

3

3

3

6

1

1

1

1

4

3

1

2

2

2

1

1

3

3

3

3

99

99

3

2

1

1

1

5

1

1

1

2

5

4

1

1

1

1

1

1

1

1

1

8

8

1

1

1

1

1

1

8

5

1

2

1

1

1

1

11

1

1

1

1

1

1

2

2

1

1

1

5

3

1

1

1

1

1

1

1

1

1

1

1

1

1

1

2

2

1

1

3

3

1

1

1

1

3

3

1

1

2

2

6

1

2

3

1

1

1

1

2

2

1

1

2

1

1

2

1

1

2

2

1

1

5

2

2

1

256

16

11

11

5

5

147

2

1

1

1

1

3

2

1

4

4

1

1

7

1

1

1

1

1

1

1

5

5

2

2

1

1

1

1

1

1

3

2

1

1

1

1

1

1

1

3

3

1

1

4

3

1

6

6

4

3

1

6

6

1

1

1

1

1

1

4

1

2

1

39

39

10

10

5

1

4

1

1

4

1

3

11

2

9

1

1

2

2

2

1

1

5

5

2

2

93

1

1

2

1

1

1

1

12

3

1

1

1

1

1

1

1

2

6

5

1

1

1

4

3

1

2

2

11

1

5

1

3

1

18

18

2

1

1

1

1

1

1

3

3

3

3

1

1

15

1

1

1

1

2

1

1

1

1

1

4

1

1

2

1

1

1

1

5

5

1

1

1

1

1

1

1

1

59

56

1

1

23

1

1

2

1

1

1

1

1

2

1

1

2

2

1

4

1

6

1

1

2

1

1

1

1

1

1

8

2

1

1

4

3

1

1

1

1

1

2

1

1

1

1

1

1

7

1

2

1

1

1

1

1

1

3

1

1

2

1

1

273

24

24

1

1

4

2

1

1

11

11

1

1

4

2

1

1

1

1

1

1

1

1

14

14

1

1

1

1

1

1

2

2

1

1

1

1

3

2

1

4

1

1

2

26

26

26

26

28

28

1

1

1

1

1

1

1

1

4

2

1

1

1

1

1

1

2

2

4

4

1

1

7

6

1

1

1

2

2

1

1

141

18

3

3

3

2

1

2

1

1

3

1

2

2

1

1

1

1

3

1

1

1

1

1

44

2

2

1

1

1

1

1

1

1

1

1

1

2

2

3

3

1

1

1

1

4

1

2

1

1

1

1

1

1

1

3

1

1

1

3

1

2

1

1

1

1

1

1

1

1

3

1

1

1

2

2

2

2

2

1

1

1

1

3

1

1

1

20

2

2

1

1

1

1

2

1

1

1

1

2

1

1

1

1

1

1

1

1

8

2

1

2

1

2

19

19

19

40

4

1

2

1

1

1

3

1

2

1

1

4

2

1

1

3

2

1

2

1

1

1

1

5

1

3

1

1

1

8

4

4

2

1

1

1

1

3

3

1

1

18

7

6

6

1

1

11

2

2

6

6

3

1

1

1

1

1

1

1

7

7

1

1

1

1

4

2

2

1

1

5

5

5

5

9

9

2

2

4

1

2

1

3

2

1

48

46

14

4

1

3

5

1

1

1

1

1

1

1

4

2

2

32

11

1

1

2

1

1

1

2

1

1

13

1

1

1

1

1

1

2

2

1

2

8

2

2

1

3

2

2

1

1

1

1

2

2

2

2

2

92

91

1

1

1

1

20

20

20

20

25

25

25

1

1

1

1

1

1

1

3

1

1

1

1

1

1

1

1

1

1

1

1

1

1

1

9

6

6

1

1

1

1

1

1

2

2

2

1

1

1

11

11

11

8

3

3

3

3

3

22

22

22

5

5

2

1

1

1

1

4

1

1

1

1

1

1

1

21

17

17

17

17

1

16

3

3

3

3

3

1

1

1

1

1

13

13

13

13

13

13

1473

755

12

12

12

12

743

7

7

7

15

15

15

5

5

5

120

57

57

5

5

1

1

5

5

6

6

11

1

10

2

2

2

2

2

2

2

2

1

1

26

5

1

1

3

6

8

1

1

24

10

10

14

12

2

214

27

26

1

2

1

1

132

50

1

1

1

1

1

9

1

2

1

2

1

1

18

1

14

1

2

4

1

15

3

1

14

9

4

1

3

3

35

1

2

1

2

1

1

1

1

1

1

1

5

1

1

1

1

1

1

1

1

1

1

1

4

1

1

1

1

5

2

2

3

3

37

1

1

8

8

6

6

2

2

4

3

1

16

10

6

21

1

1

2

2

1

1

2

1

1

4

1

1

1

1

1

1

1

1

2

2

1

1

4

3

1

1

1

1

1

65

65

1

2

2

1

1

4

1

3

1

10

3

1

1

1

7

1

1

10

1

2

1

1

4

1

2

1

1

230

8

7

1

4

4

101

2

99

1

1

5

5

2

2

29

1

28

15

1

3

2

1

1

1

6

8

1

6

1

10

2

1

7

23

1

1

3

1

1

2

1

1

1

3

3

2

2

1

19

5

1

1

1

1

8

1

1

5

2

1

2

222

222

1

1

1

1

1

1

185

1

1

1

1

1

1

2

1

1

8

8

1

1

5

2

1

2

1

1

4

1

1

1

1

1

1

1

1

2

1

1

23

1

2

1

1

1

1

1

1

1

1

1

1

1

1

1

1

1

1

2

1

1

2

1

1

1

1

2

1

1

5

1

1

1

2

3

2

1

1

1

6

1

1

1

1

1

1

1

1

1

1

5

1

1

2

1

1

1

2

1

1

1

1

1

1

1

1

3

3

41

1

1

1

2

1

1

1

3

1

1

1

1

1

2

1

1

1

2

1

1

2

1

1

2

1

1

1

1

1

1

1

1

1

1

1

1

1

1

3

1

2

1

1

1

1

1

1

3

1

1

1

1

1

2

1

1

8

1

1

1

1

1

1

1

1

1

1

1

1

1

1

3

3

3

1

2

1

1

7

1

6

7

3

1

1

2

4

1

3

1

1

1

1

3

1

2

1

1

1

1

35

5

3

2

1

1

5

5

2

2

6

6

3

3

5

1

4

3

1

2

1

1

4

4

2

2

2

2

2

288

1

1

1

1

287

99

18

1

1

1

1

1

5

1

1

2

1

1

1

1

2

2

2

1

1

47

1

3

1

2

3

2

1

11

1

2

1

2

1

1

1

1

1

1

4

1

1

2

2

1

7

3

1

3

14

1

1

5

1

1

1

1

1

1

1

6

1

1

2

2

1

1

1

1

1

1

1

1

1

47

1

1

16

1

6

2

5

1

1

14

14

14

14

1

1

1

1

4

4

2

2

136

11

6

1

1

1

2

5

5

1

1

4

1

2

1

3

1

2

21

1

14

6

3

3

1

1

13

12

1

1

1

1

1

5

3

2

13

1

12

1

1

2

2

14

3

1

2

1

6

1

14

1

12

1

4

2

1

1

1

1

2

1

1

14

1

1

12

1

1

1

1

14

14

14

14

14

158

133

33

3

2

1

3

1

1

1

1

1

1

1

2

1

1

1

1

13

12

1

1

1

4

3

1

1

1

1

1

1

1

1

1

20

7

1

2

1

1

1

1

2

1

1

1

1

3

1

2

3

3

3

1

1

1

1

1

80

1

1

1

1

20

20

2

1

1

9

1

1

1

1

1

1

1

1

1

2

2

3

1

2

2

2

6

1

1

1

2

1

1

1

2

2

1

1

6

2

4

3

3

1

1

7

7

1

1

1

1

1

1

4

1

1

2

5

1

1

1

1

1

1

1

11

11

8

8

2

2

1

1

13

13

9

2

7

3

1

1

1

1

1

1

1

1

1

6

5

5

5

5

1

1

1

1

4

4

4

4

4

8

8

8

8

8

16

16

16

16

16

24

24

24

7

7

7

17

17

1

16
