# Supplementary material for: DAIRYdb: a manually curated reference database for improved taxonomy annotation of 16S rRNA gene sequences from dairy products
Source: BMC Genomics. 2019 Jul 8;20:560. doi: 10.1186/s12864-019-5914-8 (PMC6615214; doi:10.1186/s12864-019-5914-8)
Supplement: Supplementary file 3 — Supplementary Information. Additional file 3 contains supplementary figures described in the main manuscript. It is a portable document file (pdf) that can be read with Acrobat Reader. (PDF 3925 kb) [file 12864_2019_5914_MOESM3_ESM.pdf]

Additional File 3

Figure S1

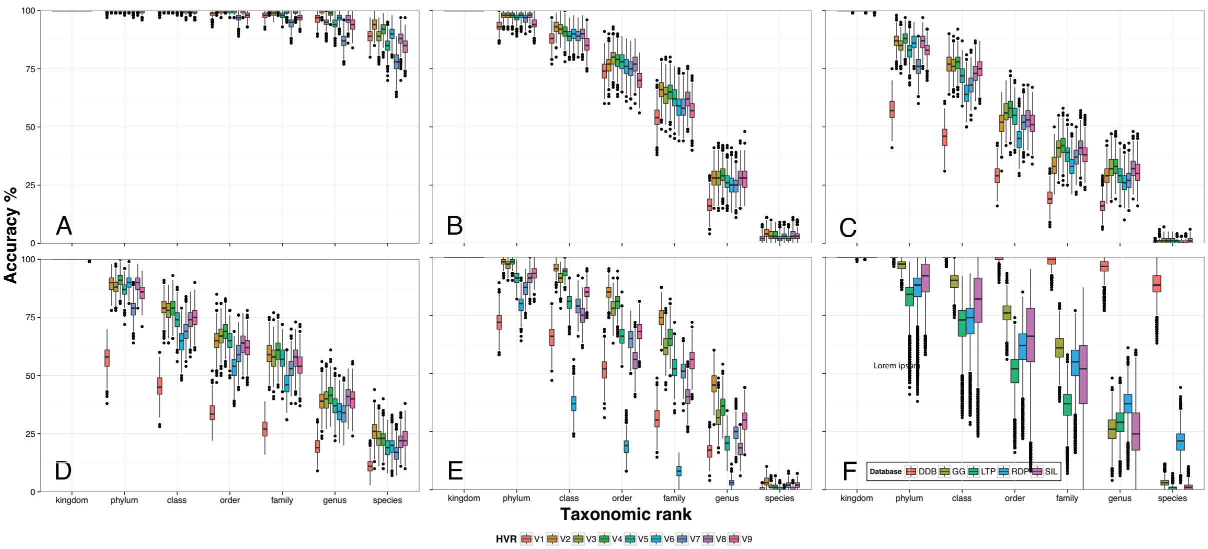

Taxonomy annotation accuracy (accuracy = correctly annotated/total) of the DAIRYdb on reads extracted with *in silico* PCR. Single HVR V1-V9 were re-annotated using SINTAX. Taxonomy annotation was bootstrapped 1000 times with a subset of 100 randomly selected sequences from the DAIRYdb and annotated with DAIRYdb (A), Greengenes (B), LTP (C), RDP (D) and Silva (E). Average performance of all HVR for each database (F).

Figure S2

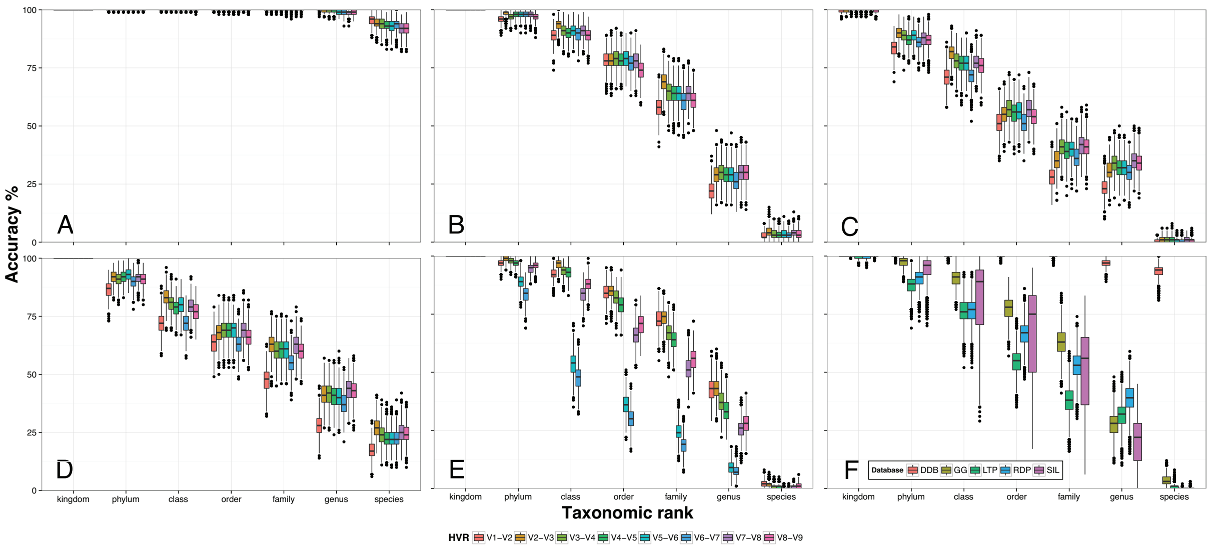

Taxonomy annotation accuracy (accuracy = correctly annotated/total) of the DAIRYdb on reads extracted with *in silico* PCR. The HVR pairs V1-V2, V2-V3, V3-V4, V4-V5, V5-V6, V6-V7, V7-V8, V8-V9 were re-annotated using SINTAX. Taxonomy annotation was bootstrapped 1000 times with a subset of 100 randomly selected sequences from the DAIRYdb and annotated with DAIRYdb (A), Greengenes (B), LTP (C), RDP (D) and Silva (E). Average performance of all HVR for each database (F).

Figure S3

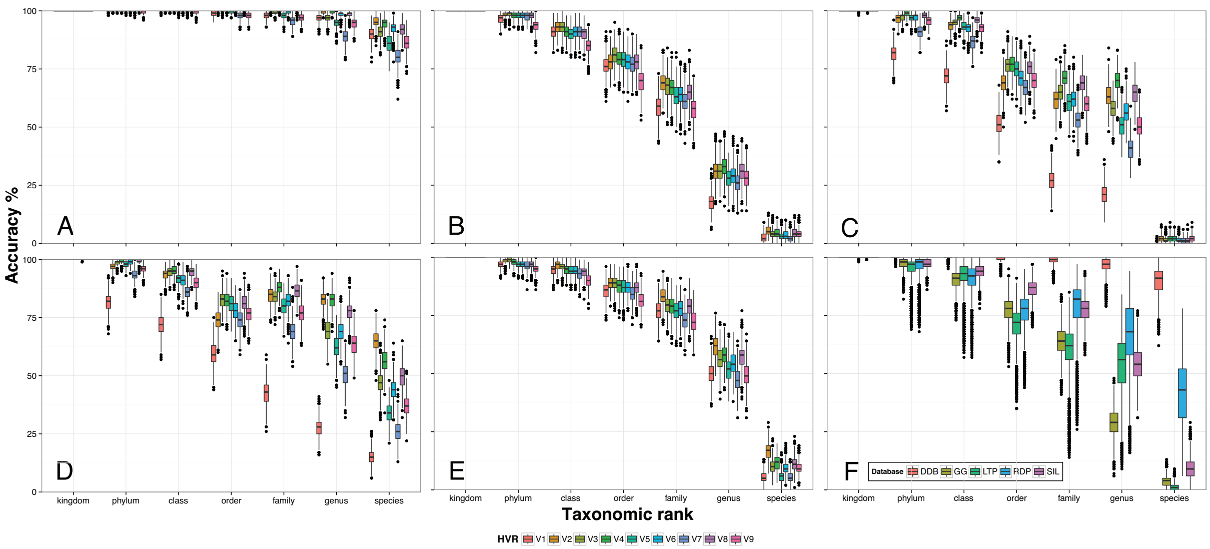

Taxonomy annotation accuracy (accuracy = correctly annotated/total) of the DAIRYdb on reads extracted with *in silico* PCR. Single HVR V1-V9 were re-annotated using Blast+. Taxonomy annotation was bootstrapped 1000 times with a subset of 100 randomly selected sequences from the DAIRYdb and annotated with DAIRYdb (A), Greengenes (B), LTP (C), RDP (D) and Silva (E). Average performance of all HVR for each database (F).

Figure S4

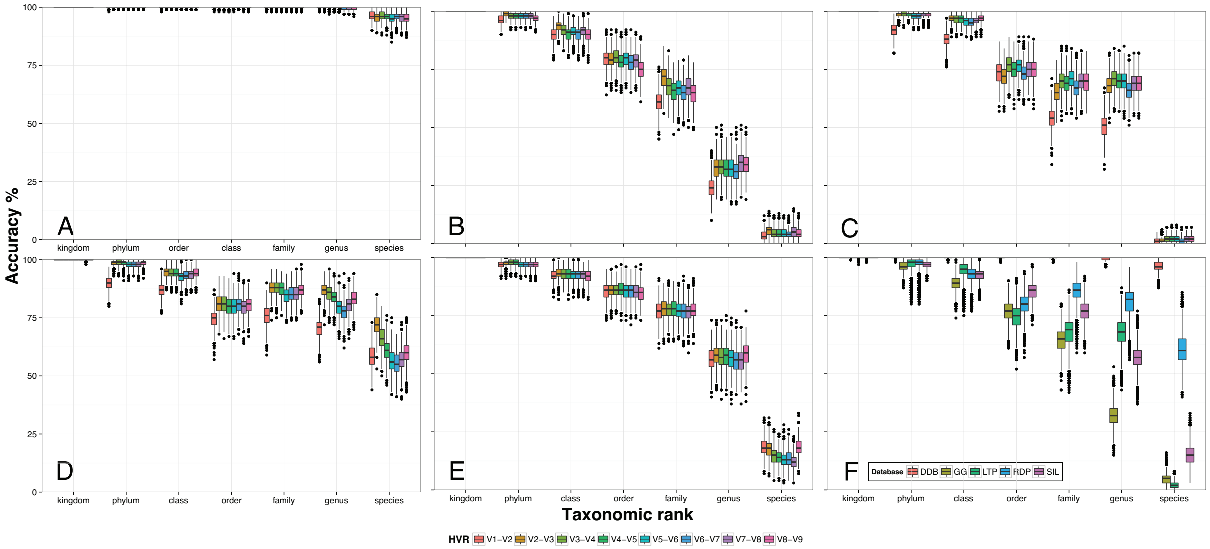

Taxonomy annotation accuracy (accuracy = correctly annotated/total) of the DAIRYdb on reads extracted with *in silico* PCR. The HVR pairs V1-V2, V2-V3, V3-V4, V4-V5, V5-V6, V6-V7, V7-V8, V8-V9 were re-annotated using Blast+. Taxonomy annotation was bootstrapped 1000 times with a subset of 100 randomly selected sequences from the DAIRYdb and annotated with DAIRYdb (A), Greengenes (B), LTP (C), RDP (D) and Silva (E). Average performance of all HVR for each database (F).

Figure S5

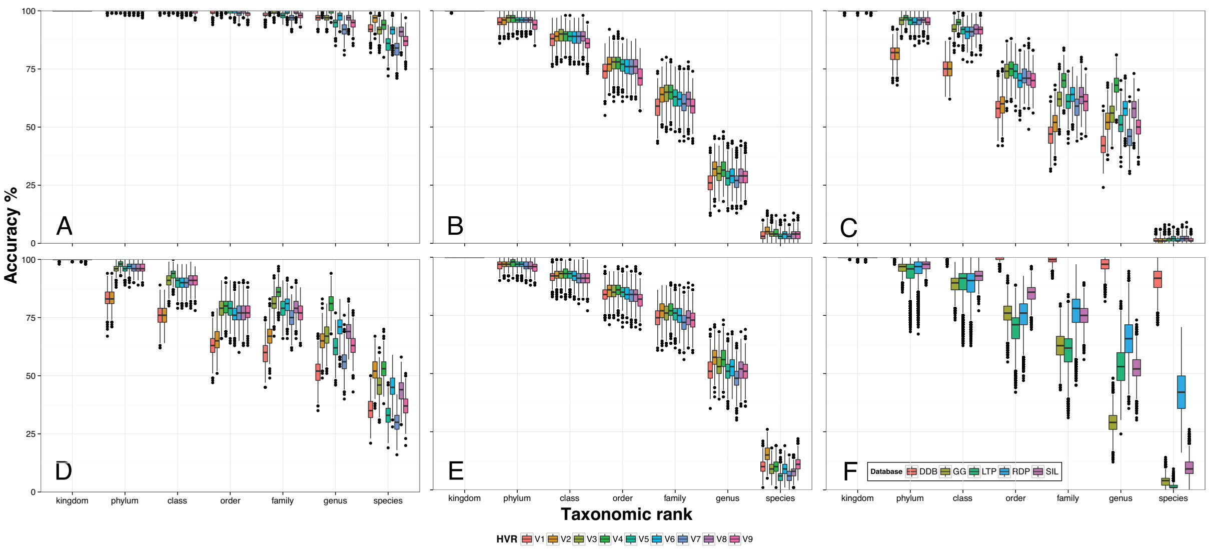

Taxonomy annotation accuracy (accuracy = correctly annotated/total) of the DAIRYdb on reads extracted with V-Xtractor. Single HVR V1-V9 were re-annotated using Blast+. Taxonomy annotation was bootstrapped 1000 times with a subset of 100 randomly selected sequences from the DAIRYdb and annotated with DAIRYdb (A), Greengenes (B), LTP (C), RDP (D) and Silva (E). Average performance of all HVR for each database (F).

Figure S6

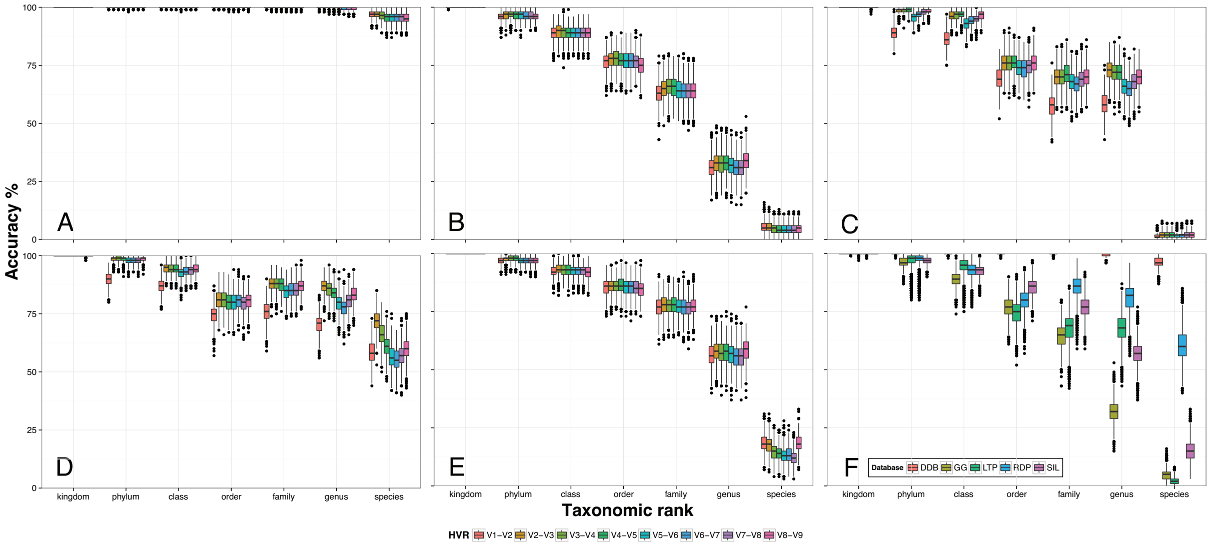

Taxonomy annotation accuracy (accuracy = correctly annotated/total) of the DAIRYdb on reads extracted with V-Xtractor. The HVR pairs V1-V2, V2-V3, V3-V4, V4-V5, V5-V6, V6-V7, V7-V8, V8-V9 were re-annotated using Blast+. Taxonomy annotation was bootstrapped 1000 times with a subset of 100 randomly selected sequences from the DAIRYdb and annotated with DAIRYdb (A), Greengenes (B), LTP (C), RDP (D) and Silva (E). Average performance of all HVR for each database (F).

Figure S7

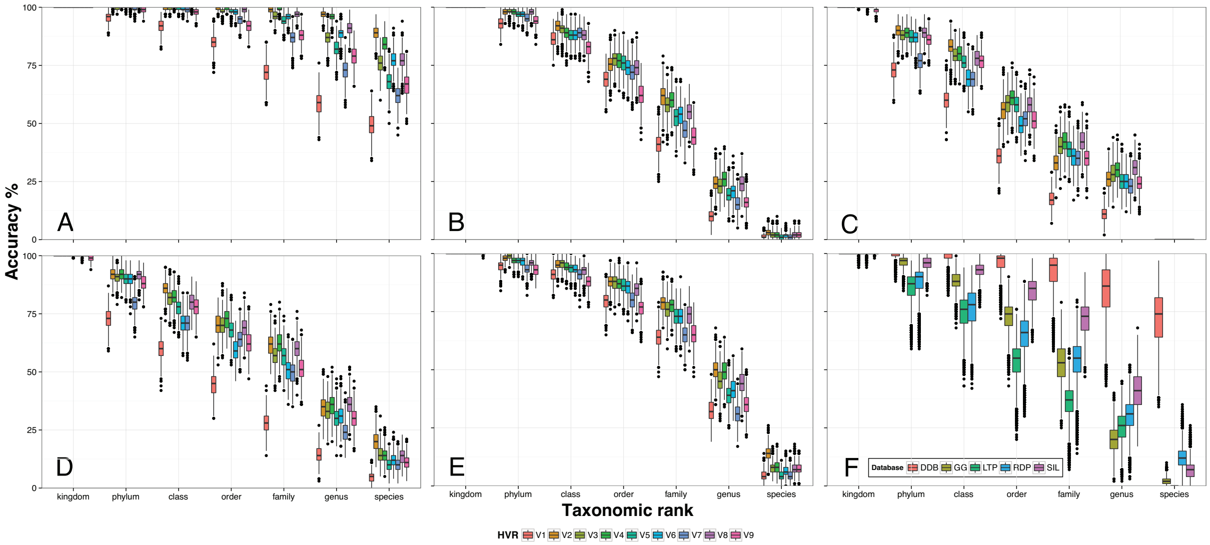

Taxonomy annotation accuracy (accuracy = correctly annotated/total) of the DAIRYdb on reads extracted with *in silico* PCR. Single HVR V1-V9 were re-annotated using Metaxa2. Taxonomy annotation was bootstrapped 1000 times with a subset of 100 randomly selected sequences from the DAIRYdb and annotated with DAIRYdb (A), Greengenes (B), LTP (C), RDP (D) and Silva (E). Average performance of all HVR for each database (F).

Figure S8

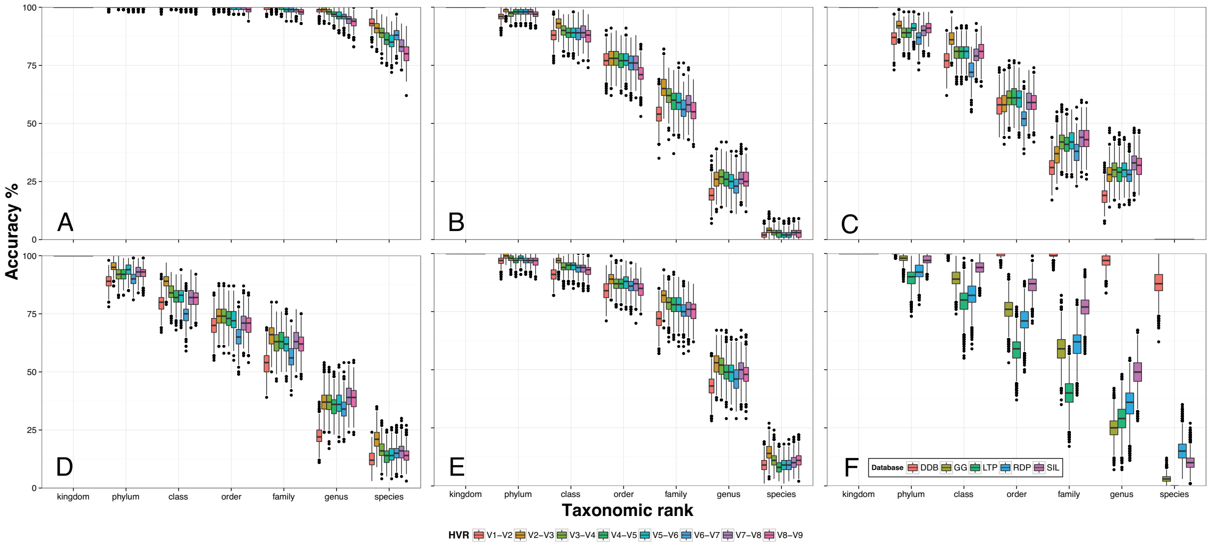

Taxonomy annotation accuracy (accuracy = correctly annotated/total) of the DAIRYdb on reads extracted with *in silico* PCR. The HVR pairs V1-V2, V2-V3, V3-V4, V4-V5, V5-V6, V6-V7, V7-V8, V8-V9 were re-annotated using Metaxa2. Taxonomy annotation was bootstrapped 1000 times with a subset of 100 randomly selected sequences from the DAIRYdb and annotated with DAIRYdb (A), Greengenes (B), LTP (C), RDP (D) and Silva (E). Average performance of all HVR for each database (F).

Figure S9

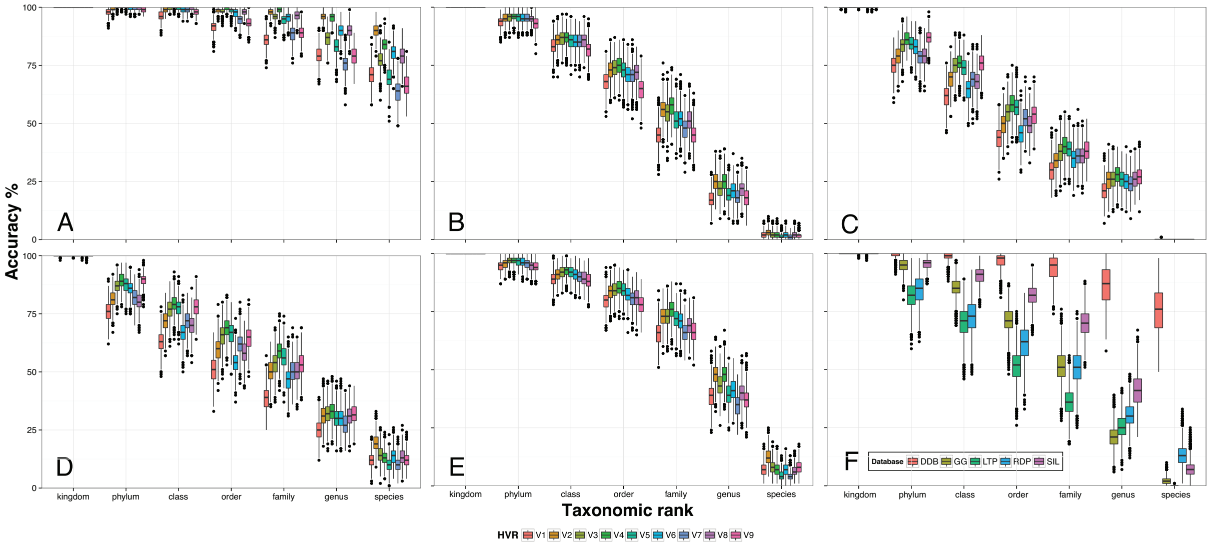

Taxonomy annotation accuracy (accuracy = correctly annotated/total) of the DAIRYdb on reads extracted with V-Xtractor. Single HVR V1-V9 were re-annotated using Metaxa2. Taxonomy annotation was bootstrapped 1000 times with a subset of 100 randomly selected sequences from the DAIRYdb and annotated with DAIRYdb (A), Greengenes (B), LTP (C), RDP (D) and Silva (E). Average performance of all HVR for each database (F).

Figure S10

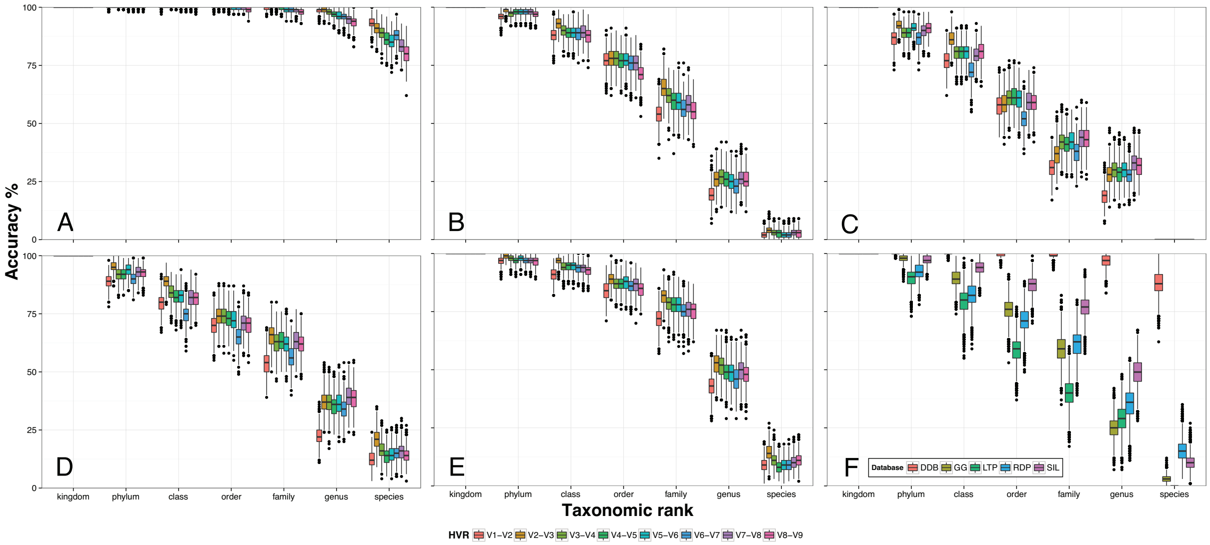

Taxonomy annotation accuracy (accuracy = correctly annotated/total) of the DAIRYdb on reads extracted with V-Xtractor. The HVR pairs V1-V2, V2-V3, V3-V4, V4-V5, V5-V6, V6-V7, V7-V8, V8-V9 were re-annotated using Metaxa2. Taxonomy annotation was bootstrapped 1000 times with a subset of 100 randomly selected sequences from the DAIRYdb and annotated with DAIRYdb (A), Greengenes (B), LTP (C), RDP (D) and Silva (E). Average performance of all HVR for each database (F).

Figure S11

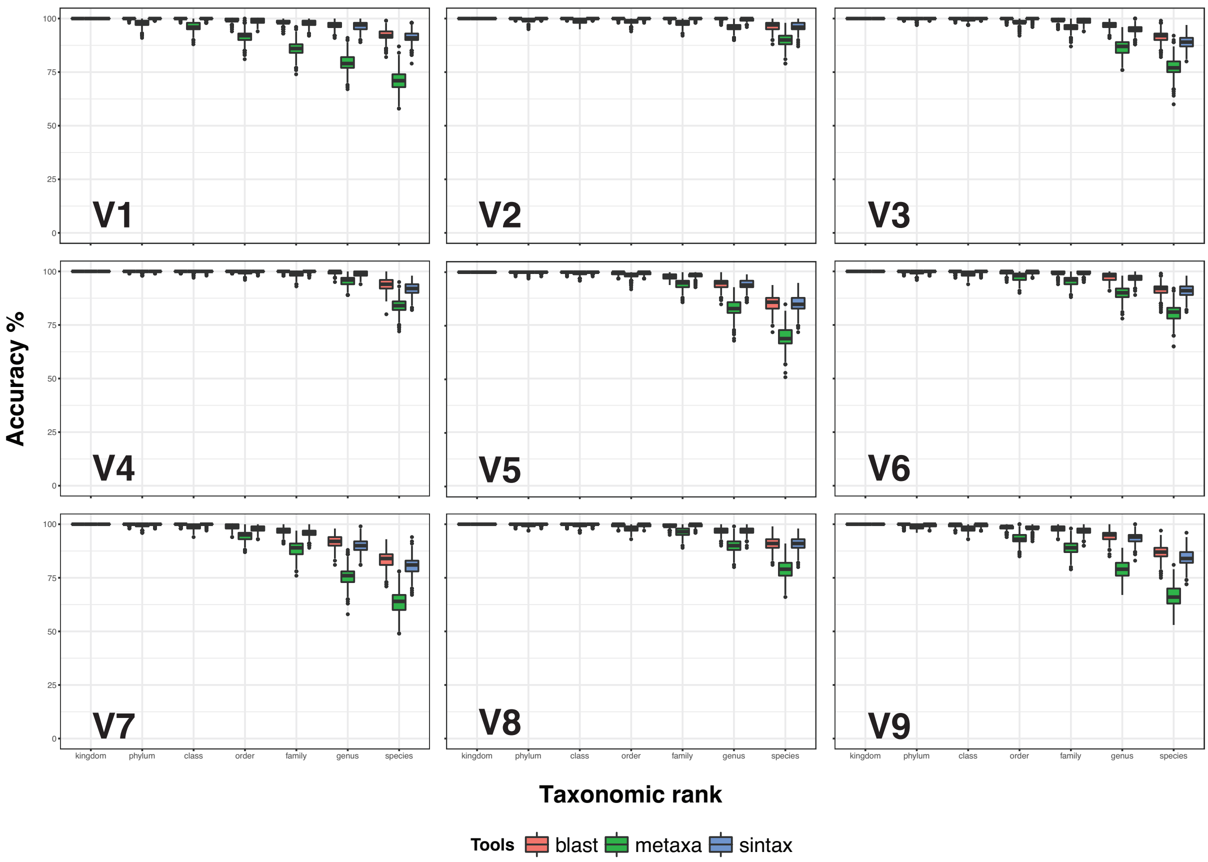

Comparison of Taxonomy annotation accuracy (accuracy = correctly annotated/total) of the DAIRYdb with different classification predictors on reads extracted with V-Xtractor. Single HVR V1-V9 were re-annotated using Blast+, Metaxa2 and SINTAX, respectively. Taxonomy annotation was bootstrapped 1000 times with a subset of 100 randomly selected sequences from the DAIRYdb.

Figure S12

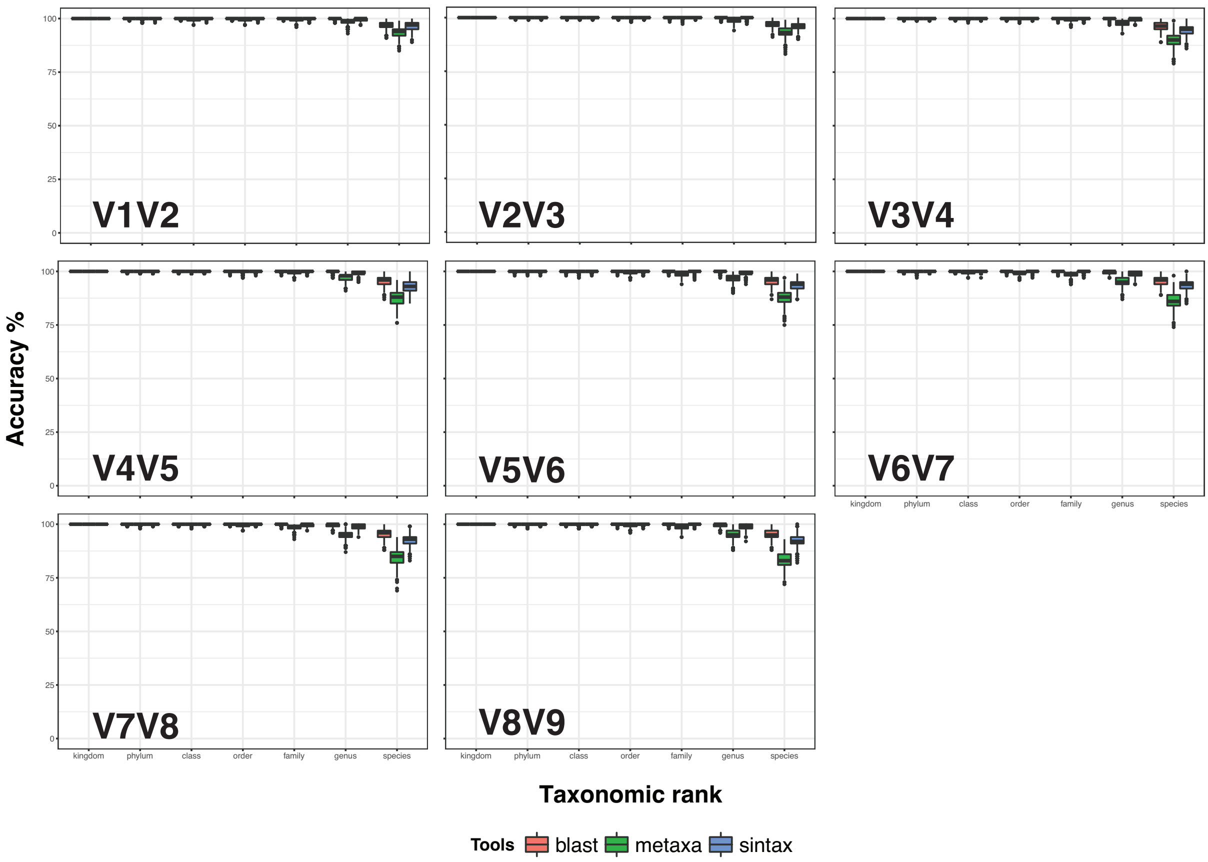

Comparison of Taxonomy annotation accuracy (accuracy = correctly annotated/total) of the DAIRYdb with different classification predictors on reads extracted with V-Xtractor. The HVR pairs V1-V2, V2-V3, V3-V4, V4-V5, V5-V6, V6-V7, V7-V8, V8-V9 were re-annotated using Blast+, Metaxa2 and SINTAX, respectively. Taxonomy annotation was bootstrapped 1000 times with a subset of 100 randomly selected sequences from the DAIRYdb.
