# Supplementary material for: DAIRYdb: a manually curated reference database for improved taxonomy annotation of 16S rRNA gene sequences from dairy products
Source: BMC Genomics. 2019 Jul 8;20:560. doi: 10.1186/s12864-019-5914-8 (PMC6615214; doi:10.1186/s12864-019-5914-8)
Supplement: Supplementary file 4 — RMarkdown for reproducibility. The additional file 4 includes the different scripts used to test all HVR primers (for single HVR and HVR pairs), scripts used to customize the different database in order to use them in the different assignation tools, and scripts used for DAIRYdb validation. We mainly used bash and R scripts [101, 102]. The file is in html format and was generated starting from a Rmarkdown file. (HTML 1012 kb) [file 12864_2019_5914_MOESM4_ESM.html]

DairyDB Validation


# DairyDB Validation

DAIRYdb: A manually curated reference database for improved taxonomy annotation of 16S rRNA gene sequences from dairy products. Marco Meola, Etienne Rifa, Noam Shani, Céline Delbes, Hélène Berthoud, Christophe Chassard

# Hyper Variable Region Extraction

The primary file `DAIRYdb_v1.1_10290_20180806_Final.fasta` contains 10290 sequences. We use two different tools to extract in silico single and couple HVR (V1 to V9 and V1V2 to V8V9). The pcr.seq function from mothur allows us to test primers performance on the sequences of the DAIRYdb. Vxtractor predicts each HVR position on the sequences, it is based on an HMM algorithm and do not depend on primer specificity.

## I. Primer performance test with Mothur pcr.seqs()

### Single HVR

Primers list:

| HVR | forward | reverse |
| --- | --- | --- |
| V1 | AGAGTTTGATCMTGGCTCAG | TTACTCACCCGTNCGCCRCT |
| V2 | AGYGGCGNACGGGTGAGTAA | GCWGCCTCCCGTAGGAGT |
| V3 | ACWCCTACGGGWGGCAGCAG | ATTACCGCGGCTGCTGG |
| V4 | GTGNCAGCMGCCGCGGTAA | GGACTACHVGGGTWTCTAAT |
| V5 | RGGATTAGATACCCY | CCGTCAATTYYTTTRAGTTT |
| V6 | AAACTYAAARRAATTGACGG | GGGTYKCGCTCGTTRY |
| V7 | RYAACGAGCGMRACCC | GAYTTGACRTCVTCCM |
| V8 | KGGABCACCGCYCGYC | GRCGRGCGGTGWGTRC |
| V9 | GYACWCACCGCYCGYC | GGNTACCTTGTTACGACTT |

Script used for the single HVR extractions with pcr.seqs function (Mothur):

```
for i in $(echo "V1 V2 V3 V4 V5 V6 V7 V8 V9""); do
echo $i

mothur "#pcr.seqs(fasta=DAIRYdb_v1.1_10290_20180806_Final.fasta,oligos=oligos/solo${i}.txt)"
mv -f DAIRYdb_v1.1_10290_20180806_Final.pcr.fasta DAIRYdb.pcr${i}_0MM.fasta

done
```

Extracted sequences counts:

| V1 | V2 | V3 | V4 | V5 | V6 | V7 | V8 | V9 |
| --- | --- | --- | --- | --- | --- | --- | --- | --- |
| 2291 | 5935 | 8566 | 9347 | 8696 | 8734 | 9045 | 7891 | 3841 |

### Couple HVR

Primers list:

| HVR | forward | reverse |
| --- | --- | --- |
| V1-V2 | AGAGTTTGATCMTGGCTCAG | GCWGCCTCCCGTAGGAGT |
| V2-V3 | AGYGGCGNACGGGTGAGTAA | ATTACCGCGGCTGCTGG |
| V3-V4 | ACWCCTACGGGWGGCAGCAG | GGACTACHVGGGTWTCTAAT |
| V4-V5 | GCCAGCAGCCGCGGTAA | CCGTCAATTYYTTTRAGTTT |
| V5-V6 | RGGATTAGATACCCY | GGGTYKCGCTCGTTRY |
| V6-V7 | AAACTYAAARRAATTGACGG | GAYTTGACRTCVTCCM |
| V7-V8 | RYAACGAGCGMRACCC | GRCGRGCGGTGWGTRC |
| V8-V9 | KGGABCACCGCYCGYC | GGNTACCTTGTTACGACTT |

Script used for the couple HVR extractions with pcr.seqs function (Mothur):

```
for i in $(echo "V1V2 V2V3 V3V4 V4V5 V5V6 V6V7 V7V8 V8V9"); do
echo $i
mothur "#pcr.seqs(fasta=DAIRYdb_v1.1_10290_20180806_Final.fasta,oligos=oligos/oligos${i}.txt)"
mv -f DAIRYdb_v1.1_10290_20180806_Final.pcr.fasta DAIRYdb.pcr${i}_0MM.fasta
done
```

Extracted sequences counts:

| V1V2 | V2V3 | V3V4 | V4V5 | V5V6 | V6V7 | V7V8 | V8V9 |
| --- | --- | --- | --- | --- | --- | --- | --- |
| 3473 | 5585 | 9068 | 8838 | 9190 | 8595 | 8081 | 3586 |

## II. HVR extraction with Vxtractor

### Single HVR

Script used for the single HVR extractions with vxtractor:

```
for i in $(echo "V1 V2 V3 V4 V5 V6 V7 V8 V9"); do
vxtractor.pl -a -i short -r $i -h /home/pri/vxtractor/vxtractor/HMMs/SSU/bacteria/ -o out${i}.fasta -c out${i}.csv DAIRYdb_v1.1_10290_20180806_Final.fasta &
done
```

Extracted sequences counts:

| V1 | V2 | V3 | V4 | V5 | V6 | V7 | V8 | V9 |
| --- | --- | --- | --- | --- | --- | --- | --- | --- |
| 9113 | 10179 | 10178 | 10187 | 10281 | 10191 | 10194 | 10206 | 6971 |  |

### Couple HVR

Script used for the couple HVR extractions with vxtractor:

```
for i in $(echo ".V1-V2. .V2-V3. .V3-V4. .V4-V5. .V5-V6. .V6-V7. .V7-V8. .V8-V9."); do
vxtractor.pl -a -i short -r $i -h /home/pri/vxtractor/vxtractor/HMMs/SSU/bacteria/ -o pairs_out${i}.fasta -c pairs_out${i}.csv DAIRYdb_v1.1_10290_20180806_Final.fasta &
done
```

Extracted sequences counts:

| V1-V2 | V2-V3 | V3-V4 | V4-V5 | V5-V6 | V6-V7 | V7-V8 | V8-V9 |  |
| --- | --- | --- | --- | --- | --- | --- | --- | --- |
| 9133 | 10103 | 10181 | 10235 | 10274 | 10177 | 10191 | 7008 |  |

# Custom Database

We carried out the customization of several existing databases (silva, rdp, greengenes, LTP) in order to assign the different HVR sequences with each of them and different assignment tools (sintax, metaxa, blast).

The different databases are available via the following links:

Greengenes 13\_5: http://greengenes.secondgenome.com/downloads/database/13\_5

RDP training set: http://drive5.com/usearch/manual10/sintax\_downloads.html

SILVA\_128\_SSURef\_Nr99\_tax\_silva.fasta.gz : https://www.arb-silva.de/no\_cache/download/archive/release\_128/Exports/

LTP123\_SSU: https://www.arb-silva.de/no\_cache/download/archive/living\_tree/LTP\_release\_123/

#### Greengenes

```
#Database and taxonomy conversion
fasta_formatter -i 97_otus_13_8.fasta -o 0GG_tab.txt -t

sort -n 0GG_tab.txt > 0GG_tab_sorted.txt

sort -n 97_otu_13_8_taxonomy.txt > 0GG_taxsorted.txt
sed "s/p__;/p__NA;/g" 0GG_taxsorted.txt|sed "s/c__;/c__NA;/g"|sed "s/o__;/o__NA;/g"|sed "s/f__;/f__NA;/g"|sed "s/g__;/g__NA;/g"|sed "s/s__$/s__NA/g" > 0GG_taxsortedNA.txt

cut -f1 0GG_tab_sorted.txt > 0GG_ID.txt
cut -f2 0GG_tab_sorted.txt > 0GG_seq.txt

#Vérif ok
cut -f1 0GG_tab_sorted.txt > temp1
cut -f1 0GG_taxsorted.txt > temp2


#Sintax
#######
cut -f2 0GG_taxsortedNA.txt|sed "s/,//g"| sed "s/k__/\;tax=d:/g"|sed "s/; p__/,p:/g"|sed "s/; c__/,c:/g"|sed "s/; o__/,o:/g"|sed "s/; f__/,f:/g"|sed "s/; g__/,g:/g"|sed "s/; s__/,s:/g"|sed "s/ //g" > sintaxIDs.txt

paste 0GG_ID.txt sintaxIDs.txt 0GG_seq.txt > temp11.txt

cat temp11.txt| awk '{print ">"$1$2"\n"$3}' > 97_otus_13_8_sintax.fasta

memrec -G -d 30 usearch -makeudb_sintax 97_otus_13_8_sintax.fasta -output 97_otus_13_8_sintax.udb -dbstep 1

#Metaxa
#######
cat 0GG_taxsortedNA.txt| sed "s/k__//g"|sed "s/; p__/;/g"|sed "s/; c__/;/g"|sed "s/; o__/;/g"|sed "s/; f__/;/g"|sed "s/; g__/;/g"|sed "s/; s__/;/g"|sed "s/ //g" > 0GG_tempTab.txt

paste 0GG_tempTab.txt 0GG_seq.txt > temp12.txt

cat temp12.txt|awk '{ print ">"$1" "$2"\n"$3}' > 0GG_temp.fasta

sed "s/ Archaea;/.A - Archaea;/" ../0GG_temp.fasta > temp1
sed "s/ Eukaryota;/.E - Eukaryota;/" temp1 > temp2
sed "s/ Bacteria;/.B - Bacteria;/" temp2 > temp1
sed "s/\.B - \(Bacteria;.*;[Mm]itochondria;\)/.M - \1/" temp1 > temp2
sed "s/\.B - \(Bacteria;.*;[Cc]hloroplast;\)/.C - \1/" temp2 > temp1
sed "s/ Unclassified;/.O - Unclassified;/" temp1 > temp2
grep ">" temp2 | cut -f 1 -d " " | rev | cut -f 1 -d "." | sort | uniq -c
sed "s/ - .*;/ - /" temp2 > temp1
yes|mv temp1 97_otus_13_8_metaxa.fasta
rm -f temp2


grep "^>" 97_otus_13_8_metaxa.fasta|cut -f1 -d" "|cut -c2- > temp01
grep "^>" ../0GG_temp.fasta|cut -f2 -d" " > temp02

paste temp01 temp02 > 97_otus_13_8_metaxa.fasta.taxonomy.txt

makeblastdb -in 97_otus_13_8_metaxa.fasta -title "97_otus_13_8_metaxa" -dbtype nucl -parse_seqids

rm -f temp*
rm -f ../0GG*

#Blast
#######
ln -s ../97_otus_13_8_sintax.fasta
makeblastdb -in 97_otus_13_8_sintax.fasta -title "97_otus_13_8_sintax" -dbtype nucl -parse_seqids
```

#### RDP training set

```
#Custom rdp training set sintax

#Sintax
#rdp_16s_v16_sp.fa is ready to use with sintax.

sed 's/;tax=d:/ /g;s/,p:/;/g;s/,c:/;/g;s/,o:/;/g;s/,f:/;/g;s/,g:/;/g;s/,s:/;/g;s/"//g;s/;$//g' rdp_16s_v16_sp.fa > rdp_16s_v16_sp_silvaMEF.fa

#METAXA
#######
out=rdp_16s_v16_sp

sed "s/ Archaea;/.A - Archaea;/" rdp_16s_v16_sp_silvaMEF.fa > temp1
sed "s/ Eukaryota;/.E - Eukaryota;/" temp1 > temp2
sed "s/ Bacteria;/.B - Bacteria;/" temp2 > temp1
sed "s/\.B - \(Bacteria;.*;[Mm]itochondria;\)/.M - \1/" temp1 > temp2
sed "s/\.B - \(Bacteria;.*;[Cc]hloroplast;\)/.C - \1/" temp2 > temp1
sed "s/ Unclassified;/.O - Unclassified;/" temp1 > temp2
grep ">" temp2 | cut -f 1 -d " " | rev | cut -f 1 -d "." | sort | uniq -c
sed "s/ - .*;/ - /" temp2 > temp1
yes|mv temp1 ${out}_metaxa.fasta
rm -f temp2

grep "^>" ${out}_metaxa.fasta|cut -c2-|cut -f1 -d" " > temp1
grep "^>" rdp_16s_v16_sp_silvaMEF.fa|cut -f2 -d" " > temp2

paste temp1 temp2 > ${out}_metaxa.fasta.taxonomy.txt

rm -f temp*

makeblastdb -in rdp_16s_v16_sp_metaxa.fasta -title "rdp_16s_v16_sp_metaxa.fasta" -dbtype nucl -parse_seqids

#Blast
######
makeblastdb -in rdp_16s_v16_sp.fa -title "rdp_16s_v16_sp.fa" -dbtype nucl -parse_seqids
```

#### SILVA128nr99

```
#Custom SILVA128nr99

in=SILVA_128_SSURef_Nr99_tax_silva.fasta
out=SILVA_128_SSURef_Nr99_tax_silva

sed "s/ /_/g" $in > 0silva_form.fasta
#Fasta to Tab
fasta_formatter -i 0silva_form.fasta -o 0silva_tab.txt -t
#Keep IDs and taxonomy
grep "^>" 0silva_form.fasta|cut -d_ -f1|sed "s/>//g" > ID_1.txt
grep "^>" 0silva_form.fasta|cut -d_ -f2- > ID_tax.txt

paste ID_1.txt 0silva_tab.txt > 0silva_tab2.txt

yes|cp 0silva_tab2.txt 0silva_tab3.txt

cat 0silva_tab3.txt|awk '{print ">"$1"\n"$3}'> 0silva_3_gg.fasta

cut -f2 0silva_tab3.txt |cut -f2- -d_ > 0silva_tax3.txt

#NA insertion, we need 7 ranks taxonomy
nli=$(wc -l 0silva_tax3.txt|cut -f1 -d" ")
for i in $(seq 1 1 $nli);do
echo ";;;;;;;";
done > temp1

paste -d"\0" 0silva_tax3.txt temp1 > temp2

sed "s/;;;;;;;/;NA;NA;NA;NA;NA;NA;/g" temp2|cut -f1-7 -d\; > 0silva_tax4.txt

paste -d\; 0silva_tax4.txt ID_1.txt > 0silva_tax5.txt

rm -f temp*

cat 0silva_tax5.txt|head

cat 0silva_tax5.txt|awk -F"\;" '{print $8"\tk__"$1"; p__"$2"; c__"$3"; o__"$4"; f__"$5"; g__"$6"; s__"$7}' > 0silva_tax4gg.txt

paste 0silva_tax4gg.txt 0silva_tab3.txt > ${out}_tab.txt
cp ${out}_tab.txt temp10.txt


###SINTAX
#########
#IDs conversion
cut -f2 temp10.txt|sed "s/,//g"| sed "s/k__/\;tax=d:/g"|sed "s/; p__/,p:/g"|sed "s/; c__/,c:/g"|sed "s/; o__/,o:/g"|sed "s/; f__/,f:/g"|sed "s/; g__/,g:/g"|sed "s/; s__/,s:/g" > sintaxIDs.txt

paste temp10.txt sintaxIDs.txt > temp11.txt

cat temp11.txt|awk -F"\t" '{print ">"$1$6";\n"$5}' > ${out}_sintax.fasta

#dbstep 2 reduced memory use
memrec -G -d 30 usearch -makeudb_sintax ${out}_sintax.fasta -output ${out}_sintax.udb -dbstep 2


###METAXA
#########
sed "s/ Archaea;/.A - Archaea;/" ../$in > temp1
sed "s/ Eukaryota;/.E - Eukaryota;/" temp1 > temp2
sed "s/ Bacteria;/.B - Bacteria;/" temp2 > temp1
sed "s/\.B - \(Bacteria;.*;[Mm]itochondria;\)/.M - \1/" temp1 > temp2
sed "s/\.B - \(Bacteria;.*;[Cc]hloroplast;\)/.C - \1/" temp2 > temp1
sed "s/ Unclassified;/.O - Unclassified;/" temp1 > temp2
grep ">" temp2 | cut -f 1 -d " " | rev | cut -f 1 -d "." | sort | uniq -c
sed "s/ - .*;/ - /" temp2 > temp1
mv temp1 ${out}_metaxa.fasta
rm -f temp2

makeblastdb -in ${out}_metaxa.fasta -title "${out}_metaxa.fasta" -dbtype nucl -parse_seqids

grep "^>" ${out}_metaxa.fasta|cut -f1 -d" "|cut -c2- > temp01
grep "^>" ../$in|cut -f2 -d" " > temp02

paste temp01 temp02 > ${out}_metaxa.fasta.taxonomy.txt

rm -f temp*


###BLAST
########
makeblastdb -in SILVA_128_SSURef_Nr99_tax_silva_sintax.fasta -title "SILVA_128_SSURef_Nr99_tax_silva_sintax.fasta" -dbtype nucl -parse_seqids
```

#### LTP

```
##LTP123
in=LTPs123_SSU.compressed.fasta
out=LTPs123_SSU

sed "s/ //g" ${in} > 0LTP_form.fasta

#Fasta to Tab
fasta_formatter -i 0LTP_form.fasta -o 0LTP_tab.txt -t

cut -f8 0LTP_tab.txt > 0LTP_seq.txt
cut -f10 LTPs123_SSU.csv > 0LTP_tax.txt
cut -f1 LTPs123_SSU.csv > 0LTP_ID.txt

#NA insertion, we need 7 ranks taxonomy
nli=$(wc -l 0LTP_tax.txt|cut -f1 -d" ")
for i in $(seq 1 1 $nli);do
echo ";;;;;;;";
done > temp1

paste -d"\0" 0LTP_tax.txt temp1 > temp2

sed "s/;;;;;;;/;NA;NA;NA;NA;NA;NA;/g" temp2|cut -f1-7 -d\; > 0LTP_tax1.txt


cat 0LTP_tax1.txt|awk -F"\;" '{print "k__"$1"; p__"$2"; c__"$3"; o__"$4"; f__"$5"; g__"$6"; s__"$7}' > 0LTP_tax1gg.txt

paste 0LTP_ID.txt 0LTP_tax1gg.txt > ${out}_qiime_tax.txt

cat 0LTP_tab.txt|awk '{print ">"$1"\n"$8}' > ${out}_qiime.fasta

ln -s qiime/${out}_qiime_tax.txt temp10.txt


###Sintax
#########
cut -f2 temp10.txt|sed "s/,//g"| sed "s/k__/\;tax=d:/g"|sed "s/; p__/,p:/g"|sed "s/; c__/,c:/g"|sed "s/; o__/,o:/g"|sed "s/; f__/,f:/g"|sed "s/; g__/,g:/g"|sed "s/; s__/,s:/g" > sintaxIDs.txt

paste 0LTP_ID.txt sintaxIDs.txt 0LTP_seq.txt > temp11.txt

cat temp11.txt|awk -F"\t" '{print ">"$1$2";\n"$3}' > ${out}_sintax.fasta

memrec -G -d 30 usearch -makeudb_sintax ${out}_sintax.fasta -output ${out}_sintax.udb -dbstep 1


###METAXA
#########
cat 0LTP_tax1.txt|awk -F"\;" '{print $1";"$2";"$3";"$4";"$5";"$6";"$6"_sp."}' > 0LTP_tax2metaxa.txt

paste 0LTP_ID.txt 0LTP_tax2metaxa.txt 0LTP_seq.txt > temp12.txt
cat temp12.txt|awk -F"\t" '{print ">"$1" "$2"\n"$3}' > ${out}_metaxa1.fasta

sed "s/ Archaea;/.A - Archaea;/" ${out}_metaxa1.fasta > temp1
sed "s/ Eukaryota;/.E - Eukaryota;/" temp1 > temp2
sed "s/ Bacteria;/.B - Bacteria;/" temp2 > temp1
sed "s/\.B - \(Bacteria;.*;[Mm]itochondria;\)/.M - \1/" temp1 > temp2
sed "s/\.B - \(Bacteria;.*;[Cc]hloroplast;\)/.C - \1/" temp2 > temp1
sed "s/ Unclassified;/.O - Unclassified;/" temp1 > temp2
grep ">" temp2 | cut -f 1 -d " " | rev | cut -f 1 -d "." | sort | uniq -c
sed "s/ - .*;/ - /" temp2 > temp1
yes|mv temp1 ${out}_metaxa.fasta
rm -f temp2


grep "^>" ${out}_metaxa.fasta|cut -f1 -d" "|cut -c2- > temp01

paste temp01 0LTP_tax2metaxa.txt > ${out}_metaxa.taxonomy.txt

rm -f temp*
rm -f 0LTP*

makeblastdb -in ${out}_metaxa.fasta -title "${out}_metaxa.fastaDB" -dbtype nucl -parse_seqids


###BLAST
########
makeblastdb -in  ${out}_sintax.fasta -title "${out}_sintax.fastaDB" -dbtype nucl -parse_seqids
```

# DairyDB Validation

## I. Assignment

All single and couple HVR extracted with pcr.seqs and vxtractor are assigned with blast/sintax/metaxa against each databases with the following command lines:

usearch9 -sintax IN.fasta -db IN.udb -tabbedout out.sintax -strand both -sintax\_cutoff 0.8

blastn -query IN.fasta -db INblastDB -num\_threads 5 -out OUT\_tax.txt -evalue 1 -outfmt 6 -perc\_identity 97 -max\_target\_seqs 1

metaxa2 -i IN.fasta -d INmetaxaDB -o OUT.metaxa –cpu 6 -T 0,75,78.5,82,86.5,94.5,98.65 -taxlevel 7 –taxonomy T

#### SINTAX on migale cluster (INRA)

```
#Databases list
../bank/silva128_99nr/SILVA_128_SSURef_Nr99_tax_silva_sintax.udb
../bank/greengenes13_8/97_otus_13_8_sintax.udb
../bank/rdp/rdp_16s_v16_sp_sintax.udb
../bank/LTPsilva123/LTPs123_SSU_sintax.udb
../bank/DAIRYdbV1/Sintax/DAIRYdb_v1.1_10290_20180806_Final.udb

#Sintax
#pcr solo

ref=../bank/DAIRYdbV1/Sintax/DAIRYdb_v1.1_10290_20180806_Final2.udb
nameref=DDB

for i in $(ls ../*fasta); do
name=$(echo $i|cut -f4 -d.|cut -c4-)
echo $name

echo -e "#$ -S /bin/bash\n#$ -cwd\n#$ -V\nusearch9 -sintax ../DAIRYdb.pcr${name}.fasta -db $ref -tabbedout out.${name}_${nameref}.sintax -strand both -sintax_cutoff 0.8\n" > ${nameref}solo_sintax_${name}.sh
qsub -q short.q -l big_mem ${nameref}solo_sintax_${name}.sh
done
```

#### BLAST on migale cluster (INRA)

```
#Databases list
../bank/DAIRYdbV1/blast/DAIRYdb_v1.1_10290_20180806_Final.fasta
../bank/blast_DB/97_otus_13_8_sintax.fasta
../bank/blast_DB/rdp_16s_v16.fa
../bank/blast_DB/LTPs123_SSU_sintax.fasta
../bank/blast_DB/SILVA_128_SSURef_Nr99_tax_silva_sintax.fasta


#pcr.seq solo
ref=../bank/blast_DB/SILVA_128_SSURef_Nr99_tax_silva_sintax.fasta
DB=SIL
for i in $(ls ../*fasta); do

name=$(echo $i|cut -f4 -d.|cut -c4-)
echo $name

echo -e "#$ -q short.q\n#$ -cwd\n#$ -V\nblastn -query ../DAIRYdb.pcr${name}.fasta -db ${ref} -num_threads 5 -out blast_${DB}_${name}_tax.txt -evalue 1 -outfmt 6 -perc_identity 97 -max_target_seqs 1" > ${DB}_blast_${name}.sh

qsub -l big_mem -pe thread 5 ${DB}_blast_${name}.sh
done
```

#### METAXA on genotoul cluster (INRA)

```
#Databases list
DDB,../bank/DAIRYdbV1/Metaxa2/blast
GG,../bank/greengenes13_8/97_otus_13_8_metaxa.fasta
LTP,../bank/LTPsilva123/LTPs123_SSU_metaxa.fasta
RDP,../bank/rdp/MEF_rdp_training_set/metaxa/rdp_16s_v16_sp_metaxa.fasta
SILVA,../bank/silva128_99nr/SILVA_128_SSURef_Nr99_tax_silva_GG_metaxa.fasta

#pcr solo

for db in $(cat ../list_DB.txt); do
ref=$(echo $db|cut -f2 -d,)
DB=$(echo $db|cut -f1 -d,)
echo $ref
echo $DB

for i in $(ls ../*fasta); do
name=$(echo $i|cut -f4 -d.|cut -c4-)
echo $name

echo -e "#$ -q workq\n/home/erifa/work/DAIRYdb/Metaxa2_2.1.3/metaxa2 -i ../DAIRYdb.pcr${name}.fasta -d ${ref} -o out.${name}_${DB}.metaxa --cpu 6 -T 0,75,78.5,82,86.5,94.5,98.65 -taxlevel 7 --taxonomy T" > ${DB}_solometaxa_${name}.sh

qsub -l h_vmem=10G -l mem=10G ${DB}_solometaxa_${name}.sh
done

done
```

## II. Assignment comparison to assess efficacy

Assignment efficacy is controlled on the output files of each assignment tools. The assignment of each sequence is compared to the curated taxonomy of DAIRYdb. Here is the script for sintax, the procedure is the same with the other tools.

```
#SINTAX quality
#Loop 1: processing of each HVR assignment files
#Loop 2: processing of each sequence
    #Taxonomy conversion to obtain comparable assignments (sed), cdb1 = curated sequence taxonomy, hvr1 = assignment of the tool/database tested.   
#Loop 3: taxonomy comparison at each taxonomic rank.
    #

for assign in $(ls *.sintax); do

for id in $(cat list_IDs.txt);do


cdb1=$(grep -m 1 -F $id DDBtax.txt|cut -f2 -d\;|sed 's/d://g;s/p://g;s/c://g;s/o://g;s/f://g;s/g://g;s/s://g;s/_/ /g'|cut -c5-)


hvr1=$(grep -m 1 -F $id $assign|cut -f4|sed 's/d://g;s/p://g;s/c://g;s/o://g;s/f://g;s/g://g;s/s://g;s/\"//g;s/_/ /g')  #-f6 pour pcr.mothur et -f4 pour vxtractor

for rank in $(seq 1 1 7); do

cdb=$(echo $cdb1|cut -f${rank} -d,)
if [[ rank -lt 7 ]]
then
hvr=$(echo $hvr1|cut -f${rank} -d,|cut -f1 -d" ")
else
hvr=$(echo $hvr1|cut -f${rank} -d,)
fi


if [[ $hvr1 == "" ]]
then
declare t_${rank}=$(echo "NA")
else

if [[ $cdb == *$hvr* ]]
then
declare t_${rank}=$(echo "1")
else
declare t_${rank}=$(echo "0")
fi
fi

if [[ $hvr1 != "" ]] && [[ $hvr == "" ]] || [[ $hvr == "NA" ]] #ou égal NA
then
declare t_${rank}=$(echo "noassign")
fi

done

echo -e "$id\t$t_1\t$t_2\t$t_3\t$t_4\t$t_5\t$t_6\t$t_7\t$hvr1\t$cdb1"

done > diff_quality/${assign}_qualityOUT.txt

done
```

```
#Output example
#1=correct assignment, 0=wrong assignment, noassign=no assignment, NA=HVR not detected.

Acidimicrobiales_Species_EU491284       NA      NA      NA      NA      NA      NA      NA              Bacteria,Actinobacteria,Acidimicrobiia,Acidimicrobiales,Acidimicrobiales Family,Acidimicrobiales Genus,Acidimicrobiales Species
Acidimicrobiales_Species_GQ246409       1       1       1       1       0       noassign        noassign        Bacteria,Actinobacteria,Acidimicrobiia,Acidimicrobiales,OM1 clade       Bacteria,Actinobacteria,Acidimicrobiia,Acidimicrobiales,Acidimicrobiales Family,Acidimicrobiales Genus,Acidimicrobiales Species
Acidimicrobiales_Species_HQ396926       1       1       1       1       0       0       noassign        Bacteria,Actinobacteria,Acidimicrobiia,Acidimicrobiales,OM1 clade,uncultured actinobacterium,NA Bacteria,Actinobacteria,Acidimicrobiia,Acidimicrobiales,Acidimicrobiales Family,Acidimicrobiales Genus,Acidimicrobiales Species
Acidimicrobiales_Species_HQ397419       1       1       1       1       0       0       noassign        Bacteria,Actinobacteria,Acidimicrobiia,Acidimicrobiales,OM1 clade,uncultured bacterium,NA       Bacteria,Actinobacteria,Acidimicrobiia,Acidimicrobiales,Acidimicrobiales Family,Acidimicrobiales Genus,Acidimicrobiales Species
Acidiphilium_angustum_D30772_TS NA      NA      NA      NA      NA      NA      NA              Bacteria,Proteobacteria,Alphaproteobacteria,Rhodospirillales,Acetobacteraceae,Acidiphilium,Acidiphilium angustum
Acidiphilium_iwatense_DQ906056  1       1       1       1       1       1       noassign        Bacteria,Proteobacteria,Alphaproteobacteria,Rhodospirillales,Acetobacteraceae,Acidiphilium      Bacteria,Proteobacteria,Alphaproteobacteria,Rhodospirillales,Acetobacteraceae,Acidiphilium,Acidiphilium iwatense
Acidiphilium_multivorum_EU8619030       1       1       1       1       1       1       noassign        Bacteria,Proteobacteria,Alphaproteobacteria,Rhodospirillales,Acetobacteraceae,Acidiphilium      Bacteria,Proteobacteria,Alphaproteobacteria,Rhodospirillales,Acetobacteraceae,Acidiphilium,Acidiphilium multivorum
```

## III. Bootstrap on assignment efficacy

diff\_bootstrap\_quality.sh allows us to carry out with 1000 bootstrap the sampling distribution of the correct assignment for each hvr/databases/assignment tool.

100 results of assignment efficacy are randomly sampled. We sum these 100 results to obtain percentage of correct assignment for each taxonomic rank. This operation is carried out 1000 times to estimate de sampling distribution of the assignment efficacy.

```
##USAGE
##diff_bootstrap_quality.sh quality_files path_to_reference HVR

#Sampling 100 quality assignment results

qualityOUT=$1
ref=$2
hvr=$3

cut -f1-8 $qualityOUT|grep -v "NA"|sed "s/noassign/0/g" > tmp_assign_${ref}_${hvr}

for k in $(seq 1 1 50);do
echo $hvr
echo $ref
echo -e "k=$k"

    for j in $(seq 1 1 20);do
    echo -e "j=$j"

        for i in $(seq 1 1 100); do

        shuf -n 1 tmp_assign_${ref}_${hvr}
        done > bootstrap_${ref}_${hvr}_${j}_${k}.txt &


    done
    wait

done


#bootstrap_ref_hvr_i_j: sample of 100 quality file results, this file is generated 1000 times.

#Each column of the bootstrap files are summed to obtain percentage of accuracy on each taxonomic rank (counts_bootstrap...txt)

#The 1000 results are concatenated, repetitions in column and ranks in row. (all_counts_ref_hvr.txt) supplementary informations like taxonomic rank and HVR are added in a new column.

#Results of each HVR are finally concatenated in a single files for each database.  (allHVRcounts_ref.txt)

#Counts
for bst in $(ls bootstrap_${ref}_${hvr}*txt); do
echo $hvr
echo $ref

cat $bst|awk -v reg="$bst" '{for (i=2;i<=NF;i++){
   sums[i]+=$i;maxi=i}
 }
 END{
   for(i=2;i<=maxi;i++){
     print(sums[i])
   }
}' > counts_${bst}

done

echo -e "${hvr}\n${hvr}\n${hvr}\n${hvr}\n${hvr}\n${hvr}\n${hvr}" > tmp_${hvr}
echo -e "kingdom\nphylum\nclass\norder\nfamily\ngenus\nspecies" > tmp_ranks_${hvr}
paste tmp_ranks_${hvr} tmp_${hvr} counts*${ref}*${hvr}*txt > all_counts_${ref}_${hvr}.txt

#cat all_counts_${ref}*.txt > allHVRcounts_${ref}.txt
```

This script is executed on each quality files (databases / HVR / assignment tool)

```
for ref in $(echo "DDB GG LTP RDP SIL"); do

for hvr in $(echo "V1 V2 V3 V4 V5 V6 V7 V8 V9"); do

echo -e "#$ -S /bin/bash\n#$ -cwd\n#$ -V\n./diff_bootstrap_quality.sh ../*${hvr}*${ref}*_qualityOUT.txt ${ref} ${hvr}" > BS_${ref}_${hvr}.sh
qsub BS_${ref}_${hvr}.sh

done
done

#When all files "all_counts..." are generated:

for ref in $(echo "DDB GG LTP RDP SIL"); do
cat all_counts_${ref}*.txt > allHVRcounts_${ref}.txt
done
```

Output example:

```
#Important file: allHVR_*.txt
#10 first column of 1000
head allHVRcounts_RDP_vxsoloblast.txt |cut -f1-10

kingdom V1  100 100 100 100 100 100 100 100
phylum  V1  80  76  77  87  86  76  81  81
class   V1  67  66  67  75  80  65  67  67
order   V1  63  55  60  65  68  57  61  66
family  V1  54  39  55  48  47  54  54  58
genus   V1  53  41  48  47  43  48  42  51
species V1  41  32  37  32  27  36  33  41
kingdom V2  100 100 100 100 100 100 99  100
phylum  V2  89  87  75  82  84  86  80  84
order   V2  80  78  70  69  75  77  74  74

tail allHVRcounts_RDP_vxsoloblast.txt |cut -f1-10

family  V8  70  67  68  68  71  69  71  63
genus   V8  64  65  66  73  68  70  65  64
species V8  42  44  45  41  44  52  45  43
kingdom V9  100 100 100 100 100 100 100 100
phylum  V9  100 97  95  94  94  92  96  97
class   V9  88  91  91  88  85  87  83  90
order   V9  78  79  74  73  70  76  76  79
family  V9  73  62  69  62  69  62  71  74
genus   V9  57  59  61  59  65  65  54  69
species V9  33  35  36  35  42  45  32  38
```

```
#Integration of a new column to differentiate databases

sed 's/^/DDB\t/g' allHVRcounts_DDB.txt > tmp_allHVRcounts_DDB.txt
sed 's/^/GG\t/g' allHVRcounts_GG.txt > tmp_allHVRcounts_GG.txt
sed 's/^/LTP\t/g' allHVRcounts_LTP.txt > tmp_allHVRcounts_LTP.txt
sed 's/^/RDP\t/g' allHVRcounts_RDP.txt > tmp_allHVRcounts_RDP.txt
sed 's/^/SIL\t/g' allHVRcounts_SIL.txt > tmp_allHVRcounts_SIL.txt

cat tmp_allHVRcounts* > TOTAL_counts.txt
rm -f tmp*
```

### Accuracy plots

With files “allHVRcounts…”, boxplots are generated to compare assignment accuracy between hvr at each rank.

```
#R boxplot ggplot2
#DDB GG LTP RDP SIL

library(ggplot2)
library(reshape2)
meth="metaxa"
meth2="vx_couple"

for(ref in c("DDB", "GG","LTP","RDP","SIL")){

#setwd("path_to_files")
A=read.table(paste("allHVRcounts_",ref,".txt",sep=""))
colnames(A)[1:2] <- c("ranks", "HVR")

variable_index=3:ncol(A)
Amelt=melt(A, 1:2, variable_index,variable.name = "iter", value.name= "Assigned_Perc")
head(Amelt)

RANK=factor(Amelt$ranks, levels(Amelt$ranks)[c(4,6,1,5,2,3,7)])


p<-ggplot(data=Amelt, aes(x=RANK,y=Assigned_Perc, fill=HVR)) + geom_boxplot() + theme_bw() + labs(x = "Taxonomic rank", y = "Accuracy %") + coord_cartesian(ylim=c(0, 100)) +theme(axis.text=element_text(size=12),axis.title=element_text(size=14,face="bold"), plot.title=element_text(size = 30), legend.position = "bottom",legend.text = element_text(size = 12), legend.title = element_text(size = 14, face = "bold"))
#print(p)

ggsave(paste("boxplot_",meth,"_",ref,"_",meth2,".eps",sep=""), width=21, height=15, units="cm")

}
```

Boxplots are generated to compare global accuracy (all HVR) of each database tested.

```
##R
A=read.table(paste("TOTAL_counts.txt",sep=""))
#A[1:100,1:10]
colnames(A)[1:3] <- c("Database", "ranks", "hvr")

A1=A[,-3]
variable_index=3:ncol(A1)
Amelt=melt(A1, 1:2, variable_index,variable.name = "iter", value.name= "Assigned_Perc")

RANK=factor(Amelt$ranks, levels(Amelt$ranks)[c(4,6,1,5,2,3,7)])

p<-ggplot(data=Amelt, aes(x=RANK,y=Assigned_Perc, fill=Database)) + geom_boxplot() + theme_bw() + labs(x = "Taxonomic rank", y = "Accuracy %") + coord_cartesian(ylim=c(0, 100)) +theme(axis.text=element_text(size=12), axis.title=element_text(size=14,face="bold"), plot.title=element_text(size = 20), legend.position = "bottom",legend.text = element_text(size = 12), legend.title = element_text(size = 10, face = "bold"))
ggsave(paste("boxplot_",meth,"_",meth2,"_total.eps",sep=""), width=21, height=15, units="cm")
```

```
#Pairwise wilcox.test
#Sampling 100times, 30 values and carry out wilcox rank test
for (i in levels(RANK)[-1]){
  iter=data.frame(row.names=1:16)
  for(j in 1:100){
    print(c(i, j))
    Bmelt = Amelt[Amelt$ranks==i,]
    perc = unlist( by(Bmelt$Assigned_Perc, Bmelt$Database,function(x){sample(x,30)} ) )
    db =rep(levels(Bmelt$Database), each = 30)

    df = cbind.data.frame(db, perc)
    res = pairwise.wilcox.test(df$perc, df$db, p.adjust.method = "bonf")
    res = melt(res$p.value, value.name = paste("iter", j,sep=""))
    
    iter = cbind.data.frame(iter, res[,3])
  }

resm = apply(iter,1,function(x){mean(as.numeric(x))})
assign( paste(i, "meanpval",sep="_"), resm )

}

Ftab_pval = na.omit( cbind(res[,1:2], phylum_meanpval, class_meanpval, order_meanpval,family_meanpval,genus_meanpval, species_meanpval) )

write.table(Ftab_pval, "fig_6F_pval.tsv", row.names=FALSE, sep="\t" )
```

- Table S1: Figure 5F adjusted p values

green = <0.001, yellow = 0.01-0.001, orange = 0.01-0.05, red = >0.05

- Table S2: Figure 6F adjusted p values

green = <0.001, yellow = 0.01-0.001, orange = 0.01-0.05, red = >0.05
